# Phenolic Compounds as Phytochemical Tracers of Varietal Origin of Some Autochthonous Apple Cultivars Grown in Serbia

**DOI:** 10.3390/molecules27217651

**Published:** 2022-11-07

**Authors:** Nikola Horvacki, Filip Andrić, Uroš Gašić, Dejan Đurović, Živoslav Tešić, Milica Fotirić Akšić, Dušanka Milojković-Opsenica

**Affiliations:** 1 Innovation Center of the Faculty of Chemistry Ltd., Studentski trg 12-16, 11000 Belgrade, Serbia; 2 Faculty of Chemistry, University of Belgrade, P.O. Box 51, 11158 Belgrade, Serbia; 3 Institute for Biological Research “Siniša Stanković”, National Institute of Republic of Serbia, University of Belgrade, 11060 Belgrade, Serbia; 4 Faculty of Agriculture, University of Belgrade, Nemanjina 6, 11080 Belgrade, Serbia

**Keywords:** *Malus domestica*, antioxidants, phytochemicals, phenolic profiles, 5-*O*-caffeoylquinic acid

## Abstract

Domesticated international (standard) apple cultivars, together with resistant apple cultivars are the core of the Serbian apple production. Furthermore, autochthonous cultivars are characterized by a good adaptability to the local environmental conditions and represent a valuable source of genetic variability, as well as an important source of the gene pool for further breeding programs. Additionally, they show a higher phenolic content and a stronger antioxidant activity, in comparison to commercial cultivars. Therefore, they are more likely to be used as a functional food. The subjects of this study were seventeen samples of fruits and leaves from autochthonous apple cultivars, five international standard cultivars, and six resistant apple cultivars. The phenolic profile was determined using ultra-high performance liquid chromatography (UHPLC), coupled with a diode array detector and a TSQ Quantum Access Max triple-quadrupole mass spectrometer. A total of twenty compounds were quantified in the samples. Most of the analyzed phenolics were detected in higher amounts in the peel, compared to the mesocarp. The results of the multivariate analysis of variance (MANOVA) indicate that 5-*O*-caffeoylquinic acid is present in the highest amount in the mesocarp, while in the peel and leaves, quercetin-glycosides were detected in the highest amount. According to the MANOVA: phloretin, phlorizin, 5-*O*-caffeoylquinic acid, kaempferol, and *p*-coumaric acid are present in significantly higher levels in the autochthonous cultivars, compared to the standard and resistant ones (in both fruits and leaves). Therefore, these compounds can be used as chemical tracers of the apple varietal origin.

## 1. Introduction

The exploitation and selection of apples (*Malus domestica* Borkh.) has been ongoing for centuries, and several thousand apple cultivars have been documented. Despite the vast diversity of cultivars available, apple production worldwide is now largely based on the cultivation of a few dozen ornamental and edible cultivars, grafted onto less than a dozen different clonal rootstocks. Usually, their maintenance and production require high levels of agrochemical measures [1]. The contents of the phenolic compounds of the new cultivars were reduced through breeding to avoid the astringent taste and rapid enzymatic browning [2]. Old and ancient apple cultivars show a higher phenolic content and stronger antioxidant activity, in comparison to commercial ones [3,4,5,6]. Subsequently, there is a necessity to consider the contribution of health-promoting compounds for the development of breeding programs, in order to counterbalance the loss of the fruit’s nutritional quality in the human diet [5]. The old and autochthonous cultivars represent an important source of the gene pool for such breeding programs, especially because the genotype has an important effect on the content of the bioactive compounds [3,7,8]. The adaptability to the environment and resistance to pests is of interest in the development of new cultivars, since there is a need for greener and safer agricultural practices and further development of organic foodstuff [9]. Autochthonous cultivars are characterized by a good adaptation to the local environmental conditions, and they represent a valuable source of genetic variability [4,8]. Consequently, they have the potential to overcome the new challenges set by the environment and climate change.

The contents of sugars, aroma compounds, phenolic compounds, and carotenoids are associated with the sensory properties of fruits, and determine their usage in industry. Additionally, these compounds are indicators of the physiological state of plants, their exposure to environmental stress, or their resistivity to the stress [7,10,11]. The industrial usage of apples as a raw material, such as in pectin production, imposes specific demands regarding apple quality [4,12].

Studying the bioactivity of functional ingredients in foodstuffs is getting more interest, especially in a sense of determination of an adequate intake for the prevention or amelioration of chronic diseases. There are several studies investigating the anti-inflammatory activity of apple phenolic compounds. Some dihydrochalcones, such as phloretin-2′-*O*-xyloglucoside and phloretin-2′-*O*-glucoside (phlorizin) have been only found in apple samples [13,14]. The phenolic compound content in apple leaves depends on the cultivar, soil, fertilization, ecological and growing conditions, the production type, time of leaves gathering, health status, and others [15,16]. According to Bonarska-Kujawa et al. [17], phloretin glycosides, phenolic acids, catechins, and some quercetin glycosides were identified as the main phenolic compounds in apple leaves. In most cases, the quantity of the polyphenols in the leaves is connected with the cultivar resistance to *Venturia inaequalis* [18]. Furthermore, apple leaves are used in traditional medicine [19].

For the adequate usage of apples as raw materials for functional food products, there is a prerequisite to identify individual compounds. Since there is still limited information about the phenolic constituents of old local apple cultivars, the aim of this study was the quantification of phenolics in seventeen autochthonous apple cultivars collected in two consecutive seasons (2018 and 2019). The aim was also to determine the compounds’ usefulness as chemical markers of varietal origin. For comparison, six apple scab (*Venturia inaequalis*) resistant cultivars and five standard apple cultivars were also collected and analyzed.

## 2. Results and Discussion

### 2.1. Spectrophotometric Antioxidant Assays of the Fruits and Leaves

The concentration of the phenolic compounds in apples is dependent on the cultivar, maturity of the fruit, conditions of the cultivation, storage, and suffered infections [20]. The total phenolic content (TPC) was determined using the Folin–Ciocalteu method, and expressed as gallic acid equivalents (GAEs), while radical scavenging activity (RSA) was determined as a degree of DPPH (2,2-Diphenyl-1-picryl-hydrazyl) quenching, and it is expressed as Trolox equivalents (TEs) [21].

The overall TPC values (Table S1, Supplementary Materials) were higher in the peel (3.13–41.6 g GAE/kg fresh weight—FW) than in the mesocarp (0.11–4.51 g GAE/kg FW). A similar finding is described by multiple authors [20,22,23]. The highest TPC value in the peel was detected in the Kadumana cultivar (autochthonous variety), sampled in 2019 (41.6 g GAE/kg FW). Furthermore, the highest TPC value in the mesocarp was detected in the Gružanjska letnja kolačara cultivar (autochthonous), sampled in 2018 (4.51 g GAE/kg FW). However, there was no significant difference in the TPC value range in the mesocarp of samples collected in two different years, according to Tukey’s honest significant difference (HSD) test at a 0.95 confidence level (Table S2, Supplementary Materials). Nevertheless, the ranges of the TPC values for the mesocarp and peel are slightly higher when compared to the data described by Jakobek and Barron [6].

In the context of a comparison of the TPC values in the mesocarp vs. the peel of the same variety, the highest ratio was found in the Jonagold cultivar (173-fold higher concentration in the peel than in the mesocarp). According to Feng et al. [24], the total phenolic content is 1.5−9.2 times higher in the apple peel than in the mesocarp. On average, in the year 2018, the TPC ratio in apple peels vs. the mesocarp was six, while in 2019, it was twenty-six. In both years, the average values of the TPC peel vs. the mesocarp ratio were higher in the standard and resistant varieties than in the autochthonous varieties (in 2018: 7.3, 8.7, and 5.1, respectively, and in 2019: 60.0, 58.1, and 17.2, respectively).

Considering all three tissue types—mesocarp, peel, and leaves, the highest levels of TPC were detected in the leaves (17.72–121.22 g GAE/kg dry weight—DW), and the maximum value was detected in the Kožara cultivar. Furthermore, the average TPC values in leaves and peels of all three variety types were significantly higher in samples from 2019, compared to 2018 (according to Tukey’s HSD test at *p* = 0.05; Table S2, Supplementary Materials).

The fruit peel and the leaves are in primary contact with unfavorable environmental factors. Consequently, their response is anticipated to be higher than in the case of the mesocarp [25]. The protective compounds tend to accumulate in the surface tissues of plants, due to their roles in the protection against ultra-violet irradiation, acting as defense chemicals against pathogens and herbivores, and also as attractants to accomplish pollination and fruit dispersal by animals [4].

When the DPPH radical scavenging activity values in the mesocarp were compared with the corresponding peels, similarly as with the TPC, the values were lower. The highest RSA value in the mesocarp was detected in the Gružanjska letnja kolačara cultivar from 2018 (45.52 mmol TE/kg FW). No statistically significant difference was observed in the RSA ranges of the mesocarp samples in the case of both the cultivar types and years (Tukey’s HSD test, *p* = 0.05; Table S3, Supplementary Materials). However, the RSA was up to fifteen times higher in the peel than in the mesocarp, in the Krtajka cultivar where it was at 124.5 mmol TE/kg in the peel sample, collected in 2019. As described by Raudone et al. [26], a notable difference in the RSA in the peel and the mesocarp is attributed to the flavonols that exhibit a better antioxidant activity in the peel. In the leaves, the highest RSA was detected in Idared, Kožara, and Williams Pride cultivars (14.0, 14.0, and 13.7 mmol TE/kg FW, respectively). The obtained RSA values for the leaves are rather comparable with the values from the mesocarp, than with the values from the peel. This is different from what was expected, based on the TPC values which are increasing, respectively, from mesocarp, via the peel to the leaf.

### 2.2. Quantification of the Individual Phenolic Compounds

Quantification was performed by ultra-high-pressure liquid chromatography (UHPLC) with a triple quadrupole mass spectrometer (MS) in the negative mode, as a detector. The quantification was supported with a diode array detector (DAD), prior to the mass spectrometer. This is a method of choice for the quantification of phenolic acids and flavonoids [3,12,14,27].

A total of twenty compounds were quantified in the samples (Table S1, Supplementary Materials). Compared to other quantified compounds in the mesocarp, 5-*O*-caffeoylquinic acid was detected in the highest amount in all mesocarp samples. The highest concentration of 5-*O*-caffeoylquinic acid (212.18 mg/kg FW) was detected in the cultivar Kopaoničanka (autochthonous cultivar), collected in 2018, and in the following year, the highest value was in the Gružanjska letnja kolačara (41.99 mg/kg FW), which in 2018 had the second highest value (206.75 mg/kg FW). This was expected, since 5-*O*-caffeoylquinic acid has previously been shown to impart some astringency, which together with the acidity, are typical characteristics of autochthonous apple cultivars [6,28]. Meanwhile, in other cultivar types, the highest concentration of 5-*O*-caffeoylquinic acid was detected in Prima, at a concentration of 134.03 mg/kg FW (resistant cultivar) and in Idared 68.37 mg/kg FW (standard cultivar). Meanwhile, the lowest concentration of 5-*O*-caffeoylquinic acid in the autochthonous cultivars was detected in Zaječarski delišes, collected in 2019 (7.22 mg/kg), which held the second lowest value in the previous year (37.20 mg/kg). Regarding all cultivar types, the lowest value of 5-*O*-caffeoylquinic acid was detected in Remura. Considering the results, described by Kschonsek et al. [2], the 5-*O*-caffeoylquinic acid concentration in the mesocarp in the standard cultivars, namely, Golden Delicious, Granny Smith, and Jonagold, are expected to be comparably low. In our study, the concentration of 5-*O*-caffeoylquinic acid in their mesocarp samples from 2018 was below 20 mg/kg FW. This is in agreement with Marks et al. [29], who proved that sweeter apples contain lower amounts of phenolics than more acid/bitter cultivars.

Phlorizin, caffeic acid, quercetin-3-*O*-rhamnoside, and quercetin-3-*O*-glucoside, were also found in high concentrations, compared to other quantified compounds in the mesocarp. The higher amount of phlorizin in the mesocarp was generally detected in samples of the autochthonous cultivars (in the range 1.76–9.00 mg/kg FW), compared to the resistant cultivars (0.34–3.87 mg/kg FW) and standard cultivars (0.46–2.87 mg/kg FW). The highest concentration was detected in cultivars Jesenji jablan and Demirka (9.00 and 8.42 mg/kg, respectively), while the lowest concentration was quantified in Remura (0.34 mg/kg in 2018 and 0.11 mg/kg FW in 2019). Quercetin-3-*O*-rhamnoside was detected in the highest amount among the flavonols in all analyzed mesocarp samples. The variety Jesenji jablan had the highest value for quercetin-3-*O*-rhamnoside (5.19 mg/kg, in 2018 and 1.04 mg/kg FW, in 2019).

In the peel, quercetin-3-*O*-glucoside was generally found in higher concentrations, compared to quercetin-3-*O*-rhamnoside, while the opposite was evident in the mesocarp. Along with the two mentioned compounds, in the peel, were also 5-*O*-caffeoylquinic acid, phlorizin and rutin, detected in higher concentrations, compared to other quantified compounds in all discussed cultivars. The detected high concentrations of quercetin glycosides in the peel, compared to their respective mesocarps, are in agreement with the results reported in other articles [12,20,23,26]. The highest concentration of 5-*O*-caffeoylquinic acid was detected in Gružanjska letnja kolačara (557 mg/kg FW), collected in 2018, while in the following year, its concentration was above the average among the autochthonous cultivars. The mentioned cultivar had the highest concentration of rutin (230 mg/kg FW) and quercetin-3-*O*-glucoside (32.31 mg/kg), in 2019, while their concentration, in 2018, was above average, regarding the other samples from the same type (variety and tissue). In 2018, Kadumana, Pamuklija, and Demirka also had concentrations of 5-*O*-caffeoylquinic acid in the peel over 400 mg/kg FW, while other autochthonous cultivars were in the range below 350 mg/kg FW. In the following season, it was detected in concentrations up to 135 mg/kg FW, in the Mionička tikvara cultivar.

Phloretin was detected in the highest amount, in leaves, up to 655 mg/kg dry weight (DW) in the Jesenji jablan cultivar, collected in 2018, and 597 mg/kg DW in Williams pride, collected in 2019. In the case of William’s Pride, the concentration of phloretin was 416 mg/kg DW in 2018, while in both years, the values for quercetin-3-*O*-glucoside were the highest, compared to other resistant cultivars. Furthermore, quercetin-3-*O*-glucoside, quercetin-3-*O*-rhamnoside and phlorizin were quantified, in most cases, in concentrations over 200 mg/kg DW. Among the mentioned compounds, phloretin has shown the widest range of concentrations, among the analyzed compounds (38.8–655.0 mg/kg DW). The maximum determined concentration of phloretin was almost eight times higher than the minimum value in the case of leaf samples in the autochthonous cultivars. The minimum value for phloretin, among all leaf samples was detected in Granny Smith (38.8 and 51.7 mg/kg DW in 2018 and 2019, respectively). In comparison to other standard cultivars, it had the maximum values for quercetin-3-*O*-rhamnoside in both years (254 and 244 mg/kg DW in 2018 and 2019, respectively).

To further discuss the obtained data, a multivariate analysis of variance (MANOVA) was used to point out the quantified compounds, as the chemical markers for the analyzed sample groups.

### 2.3. Multivariate Analysis of Variance

In order to determine the source of variation among the types of production, production years, and apple cultivars, and to consider the analyzed compounds as the indicators of the cultivar type, the MANOVA was applied. In total, 4048 concentration values were used as inputs for the MANOVA calculations, obtained as 396 concentration values (of three types of cultivars × three tissue types × two production years) measured for each of the twenty-two variables (twenty analyzed substances, TPC, and RSA values). One MANOVA run was performed. The model was used to estimate the influence of factors (*F*_1_–*F*_3_) on the phenolic content in the overall samples. The following factors and a full interaction model without quadratic terms were applied:

$$Y_{b}=b_{0}+b_{1}F_{1}+b_{2}F_{2}+b_{3}F_{3}+b_{12}F_{1}F_{2}+b_{13}F_{1}F_{3}+b_{23}F_{2}F_{3}+b_{123}F_{1}F_{2}F_{3}$$

where *Y* is the concentration of phenolics, *F*_1_ represents the three tissue types (mesocarp, peel, and leaf), *F*_2_ describes the differences among the three groups of apple cultivars (autochthonous, standard, and resistant), and *F*_3_ corresponds to the two consecutive years of production (2018 or 2019).

Since different concentrations of compounds in different plant parts are expected, the primary aim of the MANOVA was to pinpoint the combined effect of two factors—the cultivar and tissue type (plant organ). Among the analyzed compounds, twelve were able to differentiate samples statistically significantly, according to both the tissue and the cultivar type. Depending on the content of the quantified compounds in the analyzed samples, compounds were considered macro components if their average content was greater than 10 mg/kg, and micro components if their average content was lower than 10 mg/kg. Univariate tests for the significance of the factor effects of the macro components (5-*O*-caffeoylquinic acid, phloretin, phlorizin, rutin, quercetin-3-*O*-rhamnoside, and naringenin) are shown in Table 1. The results of the same significance tests for the micro components (kaempferol, protocatechuic acid, *p*-coumaric acid, ferulic acid, luteolin, and gallic acid) are shown in Table 2.

The TPC and RSA were found to be statistically significantly different, among the tissue types.

The TPC was also found to be different among the production years. According to the performed MANOVA, in the case of the sort type alone, there is no statistical difference.

The DPPH radical scavenging activity (RSA) inhibition is not considered significantly different between the samples, either according to the production year or cultivar type. Statistical parameters of the MANOVA models for the TPC and RSA values as dependent variables, are given in the Supplementary Materials (Table S4).

**Figure 1:**
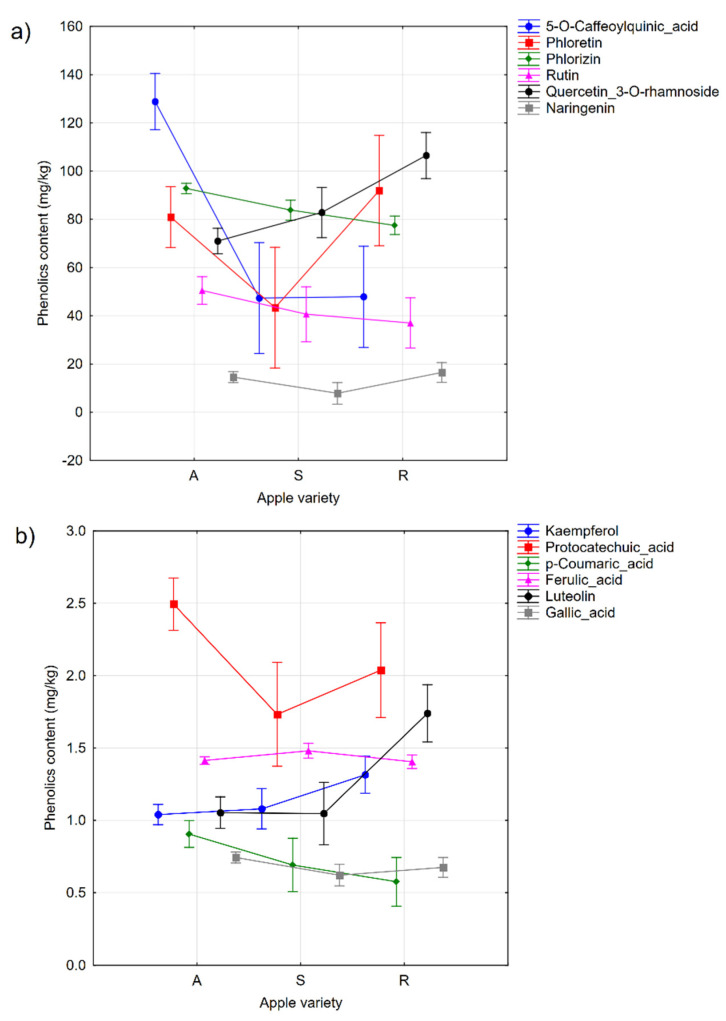
The plot of the MANOVA factor effects influencing the phenolic compound content in the samples. The phenolic content was plotted on the *y*-axis; the sort type is plotted on the *x*-axis: (A—autochthonous, S—standard, R—resistant). (**a**) phenolic macro components, and (**b**) phenolic micro components. Vertical bars denote 0.95 confidence intervals.

Considering the effect of the cultivar type, the high concentrations of 5-*O*-caffeoylquinic acid (as a phenolic macro component) and *p*-Coumaric acid (as a phenolic micro component), these are characteristic of the autochthonous cultivars (Figure 1a,b). A low phloretin concentration (as a phenolic macro component) and protocatechuic acid (as a phenolic micro component) were characteristic of the standard cultivars (Figure 1a,b). High concentrations of quercetin-3-*O*-rhamnoside (Figure 1a), as well as luteolin and kaempferol were rather characteristic of the resistant cultivars (Figure 1b).

**Figure 2:**
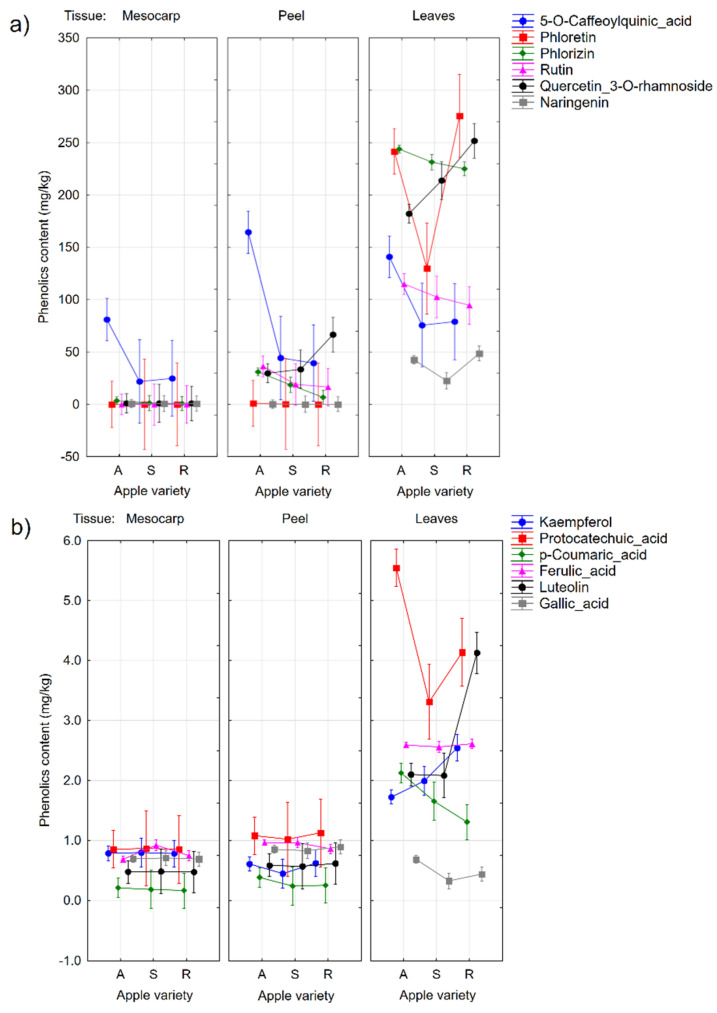
The plot of the MANOVA factor effects influencing the phenolic compound content in the different plant parts (tissues) across the different sort types. The phenolic content was plotted on the *y*-axis; the plant part and the cultivar group (A—autochthonous, S—standard, R—resistant) are plotted on the *x*-axis. (**a**) phenolic macro components, and (**b**) phenolic micro components. Vertical bars denote 0.95 confidence intervals.

Considering combined effect of the cultivar type and the plant tissue (Figure 2), in the case of the mesocarp, a statistically significant difference between the autochthonous cultivars and other variety types is observable only in the case of 5-*O*-caffeoylquinic acid.

The abundance of 5-*O*-caffeoylquinic acid and quercetin glycosides in the mesocarp is accordant to the earlier described data [4,5,23]. The mesocarps of the apple scab-resistant cultivars have lower total phenolic content values, as well as 5-*O*-caffeoylquinic acid and phlorizin concentrations, in comparison to the autochthonous cultivars. Furthermore, 5-*O*-caffeoylquinic acid and phlorizin may be more significantly affected by the harvesting year, compared to other phenolics, as suggested by a study of Belviso et al. [30]. In our study, both compounds were affected by the collection year of the cultivars (Table 1).

The peels of the autochthonous cultivars have a higher 5-*O*-caffeoylquinic acid content, compared to the resistant and standard types (Figure 2a). The peels obtained from the resistant cultivar types have a higher quercetin-3-*O*-rhamnoside and rutin (quercetin-3-*O*-rutinoside) content, compared to the autochthonous and standard ones (Figure 2a). The naringenin concentration in the leaves was higher in the autochthonous cultivars, compared to the standard and resistant cultivars. Furthermore, concentrations of phlorizin and 5-*O*-caffeoylquinic acid in the apple peel were observed to be higher in tissue infected by *V. inaequalis*, compared to the healthy tissue, as pointed out by Slatnar et al. [31]. The phlorizin content in the peel of the autochthonous cultivars is higher, compared to the resistant cultivars.

In the leaf samples of the autochthonous cultivars, the 5-*O*-caffeoylquinic acid content was higher, compared to the resistant and standard ones (Figure 2a). By the observations of Mikulic-Petkovsek et al. [16], this was expected. It is also coherent with the findings of Picinelli et al. [32] and Skłodowska. et al. [33]. Phlorizin, protocatechuic acid, *p*-coumaric, and gallic acids were also detected in higher amounts in the leaves of the autochthonous cultivars (Figure 2a,b). In the case of phloretin and naringenin, their concentrations in the standard cultivars were generally lower than in the autochthonous and resistant apple cultivars (Figure 2a). Other studies have shown that the presence of phloretin glycosides is linked to a *V. inaequalis* resistance [34], and phloretin in aglycone form is considered an active substance in the defense against *V. inaequalis* [31]. In the analyzed leaf extracts, the quercetin-3-*O*-rhamnoside content was different in all cultivar types, they were at the lowest in autochthonous cultivars, and at the highest in resistant (Figure 2a). The higher amount of quercetin-3-*O*-rhamnoside in the leaves of the resistant cultivars was also observed by Mikulic-Petkovsek et al. [16], where during the two consecutive growing seasons, the leaves of the resistant cultivars contained more quercetin-3-*O*-rhamnoside than the leaves of the susceptible cultivars. In our study, the luteolin and kaempferol concentrations in the leaves of the resistant cultivars ranged higher, compared to the other cultivars (Figure 2b).

### 2.4. Determination of the Phenolic Profile of the Mesocarp Using the UHPLC-LTQ Orbitrap MS^4^ Technique

Considering that the mesocarp is the most voluminous part of an apple, since it represents more than 90% of the whole fruit [3,25], it was important to further investigate its phenolic content, in detail, in the case of the autochthonous cultivars. Therefore, a more sophisticated and more sensitive analysis, using the UHPLC–MS/MS Orbitrap system was performed. In the analyzed mesocarp extracts, twenty phenolic acids, thirteen flavonoids, and three dihydrochalcones (their respective derivatives) were found. In Table 3, a list of the detected compounds and their fragmentations are given. Additionally, the information regarding the confirmed presence of each compound in every distinct sample is provided in the Supplementary Materials (Table S5).

Hydroxycinnamic acids were detected mainly as quinic acid esters and pentosylorhexosyl derivatives. Fragmentation occurred following the loss of sugar units 132 Da (pentose) and 162 Da (hexose) [35]. Furthermore, the quinic acid derivates were characterized by the loss of 162 Da units [36]. In the MS^2^ spectrum, the 179 *m/z* and 163 *m/z* fragments were characteristic for caffeic and *p*-coumaric acid derivates, while in the MS^3^ spectrum, the 135 m/z and 119 *m/z* fragments were remnants of the corresponding constituent acid in the MS^2^ spectrum and fragments derived by further decarboxylation in the MS^3^ spectrum [36,37]. Most of the detected compounds were previously described in apples by multiple authors [3,24,38,39,40]. Compounds **15**, **17**, and **18**, assigned as methyl *p*-coumaroylquinates, are also found in woodruff (*Galium odoratum*) [37], strawberry (*Fragaria x ananassa* Duch.), and blueberry fruits (*Vaccinium corymbosum* L.) [35]. In cider apples, methyl esters of *p*-coumaroylquinic and *p*-caffeoylquinic acid were previously detected by Sanoner et al. [41]. Compound **20** produced an MS^2^ 367 *m/z* base peak, which further, in MS^3^ gave a 179 *m/z* base peak. A 161 *m/z* peak was also observed. These peaks can be assumed as decarboxylated remnants of caffeic and ferulic acid. Therefore, compound **20** was identified as caffeoyl-feruloylquinic acid [37,42].

Phloretin and its glycosylated derivates were found—phlorizin (hexoside) and phloretin-2′-*O*-(2′′-*O*-pentosylhexoside). A 273 *m/z* base peak in the MS^2^ spectrum (parent ion in the case of phloretin) was characteristic of phloretin glycosides. In further degradation, in the MS^3^ spectrum, the 167 *m*/*z* base peak was characteristic (same base peak in MS^2^ for phloretin).

Regarding the flavonoids, three procyanidin B type dimers were detected (compounds **23**, **25**, and **27**). Along with the 425 *m/z* base peak in the MS^2^ spectrum, the presence of a 289 *m/z* peak was evident as a consequence of the loss of one flavan-3-ol unit [3,43]. In the case of the detected catechins (compounds **26** and **28**), an MS^2^ base peak at 245 *m/z* was found, while their glycoside (compound **22**) was detected, as the mentioned fragment in the MS^3^ spectrum, as a base peak [39,44]. Compound **24**, with the parent ion at 425.0878 *m/z*, was identified as epiafzelechin-3-*O*-gallate, which is described in tea (*Camellia sinensis*) cultivars [45]. Compound **29** was identified as (epi)catechin-methyl(epi)gallocatechin [43]. Other flavonoids (compounds **30**–**33**) were described, according to the previously reported mass spectrometry fragmentation rules [36].

## 3. Materials and Methods

### 3.1. Plant Material

The samples (Table 4) were collected at the Experimental Station Radmilovac, University of Belgrade, Faculty of Agriculture. The fruits were collected in August, September, and October of 2018 and 2019, at the corresponding physiological maturity of each cultivar. The leaves were collected in July of 2018 and 2019, when the photosynthetic activity was at its highest. Apple trees were under the standard cultural practice. For each cultivar, the fruits and leaves were sampled from five trees. From the orchard, the fruits and leaves were taken in a refrigerator to the laboratory and frozen until the preparation of the samples.

### 3.2. Reagents and Standards

Acetonitrile, formic acid (both MS grade), Folin–Ciocalteu reagent, sodium carbonate, and hydrochloric acid were purchased from Merck (Darmstadt, Germany) and methanol (HPLC grade) was purchased from Avantor (Gliwice, Poland). Quercetin-3-*O*-glucoside, quercetin-3-*O*-rhamnoside, isorhamnetin-3-*O*-glucoside, isorhamnetin-3-*O*-rutinoside, and kaempferol-7-*O*-glucoside were purchased from Extrasynthese (Genay, France). Trolox (6-hydroxy-2,5,7,8-tetramethylchroman-2-carboxylic acid), 2,2-Diphenyl-1-picryl- hydrazyl (DPPH), and the rest of the phenolic compound standards listed in Table S1 were purchased from Sigma Aldrich (Steinheim, Germany). Ultra-pure water (Thermo Fisher TKA MicroPure water purification system, 0.055 mS/cm) was used to prepare the standard solutions and blanks. Syringe filters (13 mm, PTFE membrane 0.45 mm) were purchased from Supelco (Bellefonte, Pennsylvania).

### 3.3. Extraction Procedures

Following the collection, the mesocarp and the apple peel were separated. The peel and the mesocarp were separately stored in a freezer. The frozen sample material was chopped in a kitchen blender. An aliquot (about 100 g for the mesocarp, and 10 g for the peel) was homogenized and further stored in a freezer at 20 °C [5,16,46].

Approximately 2.5 g of the homogenate was extracted. The extraction was performed with 25 mL of acidified methanol (0.1% HCl), with exposure to ultrasound for 1 h, similarly, as earlier described by Pavlović et al. [46]. The obtained mixture was centrifuged at 12,000 rpm for 15 min. The supernatants were stored in a freezer until further analysis.

The collected leaves were dried at room temperature in a laminar airflow, for 5 days. The extraction was similarly performed—with 10 mL of the acidified mixture (0.1% HCl) of methanol and water (70:30 *v*/*v*), with exposure to ultrasound for 1 h. About 0.5 g of ground leaf sample was measured for extraction. The extraction was conducted, as earlier described by Fotirić Akšić et al. [47]. The obtained mixture was centrifuged at 9000 rpm for 15 min. The supernatants were also stored in a freezer until further analysis.

### 3.4. Spectrophotometric Tests

#### 3.4.1. Total Phenolic Content (TPC) Determination

An aliquot of diluted sample extracts, 0.5 mL, and 0.5 mL ultrapure water were mixed with 2.0 mL of the diluted Folin–Ciocalteu (10% *v*/*v*) reagent. Then, after 5 min, 2.5 mL of 7.5% sodium carbonate was added. The blank was prepared similarly, instead of the 0.5 mL diluted sample extract and 0.5 mL ultrapure water, 1.0 mL ultrapure water was used with the mentioned reagents. The mixture was left to stand for 2 h and the absorbance was measured at 765 nm, using a GBC Cintra 6 UV–Visible spectrophotometer. The calibration curve was constructed using gallic acid standard solutions (in the range of 20–100 ppm). The results were expressed as the grams of gallic acid equivalents (GAEs) per kg of the sample [21,47,48].

#### 3.4.2. Radical Scavenging Activity (RSA) Determination

An amount of 0.1 mL of the diluted extracts was mixed with 4 mL of 79 µM DPPH (2,2-diphenyl-1-picrilhydrazil) solution in methanol and then left to stand for 60 min in the dark. The reduction of the DPPH radical was measured by the decrease of absorption at 517 nm, in reference to the blank (DPPH solution, mixed with methanol in the same amount as the analyzed extract). The RSA was calculated as a percentage of the DPPH discoloration. The results were obtained as the concentration of mmol equivalents of Trolox which correspond to the sample [21,46].

### 3.5. UHPLC–DAD MS/MS Analysis of Phenolic Compounds

The separation and quantification of the components in the samples were performed using a Dionex Ultimate 3000 UHPLC system, equipped with a diode array detector (DAD) that was connected to a TSQ Quantum triple-quadrupole mass spectrometer (UHPLC-DAD MS/MS). The list of quantified compounds is given in Table S1 (Supporting information).

The elution was performed at 40 °C on a Syncronis C18 column (100 × 2.1 mm, 1.7 μm particle size, Thermo Fisher Scientific). The mobile phase consisted of water and 0.1% formic acid (component A) and acetonitrile (component B). The flow rate was set to 0.4 mL/min, with elution in the gradient mode. The heated electrospray ionization (HESI) source was operated in the negative mode. The injection volume was 5 µL. Detection wavelengths were set to 254 nm and 280 nm. The applied elution gradient and HESI source parameters were set, as described by Gašić et al. [27].

Xcalibur software 2.2 (Thermo Fisher, Bremen, Germany) was used for the instrument control. The phenolics were identified by direct comparison with the commercial standards. The quantitative analysis was performed using two MS^2^ fragments for each compound that were previously defined as dominant. The total amounts of each compound were evaluated by calculation of the peak areas and are expressed as mg/kg.

### 3.6. UHPLC—LTQ Orbitrap MS^4^

Separation of the compounds of interest was performed using a liquid chromatography system that consisted of a quaternary Accela 600 pump and an Accela Autosampler, connected to a linear ion trap–orbitrap hybrid mass spectrometer (LTQ OrbiTrap XL) with a heated electrospray ionization probe, HESI-II (Thermo Fisher Scientific, Bremen, Germany).

A Syncronis C18 column (100 × 2.1 mm, 1.7 μm particle size, Thermo Fisher Scientific) was used as the analytical column for the separation. The mobile phase consisted of water + 0.1% formic acid (A) and acetonitrile + 0.1% formic acid (B). A linear gradient program at a flow rate of 0.300 mL/min was used. The injection volume was 5 µL. The mass spectrometer was operated in the negative ion mode. The applied elution gradient and HESI source parameters were set, as described by Gašić et al. [49].

Xcalibur software 2.1 (Thermo Fisher, Bremen, Germany) was used for the instrument control, data acquisition, and data analysis. The phenolics were identified according to the corresponding spectral characteristics (mass spectra, accurate mass, characteristic fragmentation, and characteristic retention time). In order to detect the monoisotopic mass of the unknown compounds, a full scan analysis was employed, while the fragmentation pathway was obtained by MS/MS. This exact mass search method was based on a high resolution MS analysis (Orbitrap), an online database search, and prediction of the MS/MS fragmentation using Mass Frontier 6.0 software (Thermo Fisher Scientific) [35].

### 3.7. Statistical Analysis

The multivariate analysis of variance (MANOVA) was performed, in order to estimate the differences between the cultivars and sample types, depending on the overall variability in the content of the phenolics. It was carried out using the general linear module, a part of the Statistica software (Statistica v.10, Statsoft Inc. Tulsa, OK, USA). The concentrations of the phenolics were used as dependent variables. The cultivar, part of a plant, (mesocarp, peel, or leaf), and years of production were used as categorical variables.

## 4. Conclusions

This research comprehensively investigated the total phenolic content, antioxidant capacity, and phenolic composition in Serbian autochthonous apple cultivars and compared them to the standard and resistant ones. The selected standard and resistant varieties are being widely used in commercial apple production. Therefore, this research tried to emphasize the differences between the local varieties, compared to the known varieties presented in worldwide agricultural practice. 5-*O*-caffeoylquinic acid, phlorizin, and phloretin, in both the mesocarp and the peel, as well as quercetin-glycosides in the peel, were detected as the most abundant phenolic compounds in autochthonous apple cultivars, compared to the standard and resistant ones.

The leaves of the autochthonous cultivars stood out due to the high levels of rutin, 5-*O*-caffeoylquinic acid, and naringenin.

Based on these results phloretin, phlorizin, naringenin, 5-*O*-caffeoylquinic acid (as phenolic macro components), and kaempferol, *p*-coumaric acid, gallic acid, and protocatechuic acid (as phenolic micro components) can be used to discriminate the local cultivars from the standard and resistant ones. Therefore, these compounds can be considered as chemical tracers of their varietal origin.

Additionally, the remarkable impact of the genetic features was noticed in the content of the phenolic compounds. In many facets, the autochthonous cultivars demonstrated a superiority over the standard and resistant ones. In that sense, considering the higher content of 5-*O*-caffeoylquinic acid, phloretin, and phlorizin as the desirable features from a nutritional and health-preserving perspective, the fruits and leaves of autochthonous apple cultivars can be recommended as a good source of bioactive compounds.

Further genetic investigations are required, in order to determine the comprehensive relationships between the phenolic profiles and the genetic features in the apple cultivars.

## Figures and Tables

**Table 1:** Univariate test for the significance of the factor effects influencing the phenolic macro-component levels in the analyzed samples. *DF*—Degrees of freedom; *SSs*—Sum of squares; *MSs*—Mean squares. Factors: *F*_1_ represents the three tissue types (mesocarp, peel, and leaf), *F*_2_ describes the differences among the three apple cultivars (autochthonous, standard, and resistant), and *F*_3_ corresponds to the years of production. Statistically significant results at *p* = 0.05 are denoted with *.

		**5-*O*-Caffeoylquinic Acid**				**Phloretin**				**Phlorizin**			
**Factor**	***DF***	***SS***	***MS***	***F***	***p***	***SS***	***MS***	***F***	***p***	***SS***	***MS***	***F***	***p***
Intercept	1	721,442	721,442	176.92	<0.001 *	671,792	671,792	138.98	<0.001 *	927,691	927,691	6835	<0.001 *
*F*_1_	2	71,823	35,911	8.81	<0.001 *	1.33 × 10^6^	667,048	138.00	<0.001 *	1.44 × 10^6^	718,284	5292	<0.001 *
*F*_2_	2	278,880	139,440	34.19	<0.001 *	43,735	21,867	4.52	0.012 *	7188	3594	26.48	<0.001 *
*F*_3_	1	44,856	44,856	11.00	<0.001 *	171.45	171	0.035	0.851	1918	1918	14.13	<0.001 *
*F*_1_ × *F*_2_	4	36,913	9228	2.26	0.065	87,107	21,777	4.50	0.002 *	2474	618	4.56	0.002 *
*F*_1_ × *F*_3_	2	49,047	24,523	6.01	<0.001 *	420.37	210	0.04	0.957	4113	2056	15.15	<0.001 *
*F*_2_ × *F*_3_	2	61,802	30,901	7.58	<0.001 *	1063	531	0.11	0.896	45	22	0.17	0.846
*F*_1_ × *F*_2_ × *F*_3_	4	90,284	22,571	5.54	<0.001 *	2237	559	0.12	0.977	638	159	1.18	0.323
		**Rutin**				**Quercetin-3-*O*-Rhamnoside**				**Naringenin**			
**Factor**	***DF***	***SS***	***MS***	***F***	***p***	***SS***	***MS***	***F***	***p***	***SS***	***MS***	***F***	***p***
Intercept	1	236,066	236,066	236.66	<0.001 *	973,440	973,440	1163	<0.001 *	21,776	21,776	141	<0.001 *
*F*_1_	2	256,056	128,028	128.35	<0.001 *	1.12 × 10^6^	558,657	667.70	<0.001 *	40,385	20,192	130	<0.001 *
*F*_2_	2	6105	3052	3.06	0.050 *	34,919	17,459	20.87	<0.001 *	1408	704	4.55	0.012 *
*F*_3_	1	13,487	13,487	13.52	<0.001 *	1686	1686	2.01	0.158	0.226	0.226	0.00	0.969
*F*_1_ × *F*_2_	4	3128	782	0.78	0.537	24,235	6058	7.24	<0.001 *	2806	701	4.54	0.002 *
*F*_1_ × *F*_3_	2	7595	3797	3.81	0.024 *	7593	3796	4.54	0.012 *	10.7	5.34	0.03	0.966
*F*_2_ × *F*_3_	2	967	483	0.49	0.617	2029	1014	1.21	0.300	21.8	10.9	0.07	0.932
*F*_1_ × *F*_2_ × *F*_3_	4	2352	588	0.59	0.671	1775	443	0.53	0.713	51.2	12.8	0.08	0.988
		**Quercetin**				**Quercetin-3-*O*-glucoside**							
**Factor**	***DF***	***SS***	***MS***	***F***	***p***	***SS***	***MS***	***F***	***p***				
Intercept	1	45,014	45,014	311.0	<0.001 *	2.78 × 10^6^	2.78 × 10^6^	2630	<0.001 *				
*F*_1_	2	21,784	10,892	75.25	<0.001 *	2.68 × 10^6^	1.34 × 10^6^	1267	<0.001 *				
*F*_2_	2	18.4	9.18	0.06	0.939	3056	1528	1.45	0.239				
*F*_3_	1	200	200	1.38	0.241	46,937	46,937	44.39	<0.001 *				
*F*_1_ × *F*_2_	4	749	187	1.29	0.275	6876	1719	1.63	0.170				
*F*_2_ × *F*_3_	2	2973	1486	10.27	<0.001 *	73,995	36,997	34.99	<0.001 *				
*F*_2_ × *F*_3_	2	31.6	15.8	0.11	0.897	628	314	0.30	0.743				
*F*_1_ × *F*_2_ × *F*_3_	4	329	82.3	0.57	0.686	2525	631	0.60	0.665				

**Table 2:** Univariate test for the significance of the factor effects influencing the phenolic micro-components level in the analyzed samples. Statistically significant results at *p* = 0.05 are denoted with *.

		***p*-Coumaric Acid**				**Protocatechuic Acid**				**Caffeic Acid**			
**Factor**	***DF***	***SS***	***MS***	***F***	***p***	***SS***	***MS***	***F***	***p***	***SS***	***MS***	***F***	***p***
Intercept	1	67.86	67.86	258.70	<0.001 *	564.05	564.05	571.60	<0.001 *	139.29	139.29	380.14	<0.001 *
*F*_1_	2	61.25	30.63	116.76	<0.001 *	326.88	163.44	165.62	<0.001 *	69.83	34.91	95.29	<0.001 *
*F*_2_	2	3.48	1.74	6.63	0.002 *	16.49	8.24	8.35	<0.001 *	1.27	0.64	1.73	0.180
*F*_3_	1	0.03	0.03	0.12	0.730	7.05	7.05	7.15	0.008 *	0.10	0.10	0.28	0.599
*F*_1_ × *F*_2_	4	3.67	0.92	3.49	0.009 *	32.37	8.09	8.20	<0.001 *	4.44	1.11	3.03	0.019 *
*F*_1_ × *F*_3_	2	6.20	3.10	11.82	<0.001 *	82.05	41.02	41.57	<0.001 *	7.74	3.87	10.56	<0.001 *
*F*_2_ × *F*_3_	2	0.26	0.13	0.50	0.604	0.95	0.48	0.48	0.617	5.65	2.82	7.71	<0.001 *
*F*_1_ × *F*_2_ × *F*_3_	4	0.12	0.03	0.11	0.977	1.40	0.35	0.35	0.841	2.56	0.64	1.75	0.142
		**Ferulic Acid**				**Gallic Acid**				**Luteolin**			
**Factor**	***DF***	***SS***	***MS***	***F***	***p***	***SS***	***MS***	***F***	***p***	***SS***	***MS***	***F***	***p***
Intercept	1	265.75	265.75	13,086	<0.001 *	59.81	59.81	1415	<0.001 *	211.86	211.86	588.07	<0.001 *
*F*_1_	2	86.45	43.23	2128	<0.001 *	3.06	1.53	36.18	<0.001 *	144.00	72.00	199.85	<0.001 *
*F*_2_	2	0.13	0.06	3.09	0.048 *	0.41	0.21	4.89	0.009 *	13.68	6.84	18.99	<0.001 *
*F*_3_	1	21.29	21.29	1048	<0.001 *	1.77	1.77	41.83	<0.001 *	0.01	0.01	0.03	0.863
*F*_1_ × *F*_2_	4	0.43	0.11	5.32	<0.001 *	0.95	0.24	5.63	<0.001 *	26.20	6.55	18.18	<0.001 *
*F*_1_ × *F*_3_	2	12.43	6.22	306.09	<0.001 *	6.74	3.37	79.76	<0.001 *	8.50	4.25	11.79	<0.001 *
*F*_2_ × *F*_3_	2	0.26	0.13	6.34	0.002 *	0.04	0.02	0.47	0.627	0.59	0.30	0.82	0.441
*F*_1_ × *F*_2_ × *F*_3_	4	0.19	0.05	2.28	0.063	0.04	0.01	0.21	0.933	1.39	0.35	0.96	0.430
		**Apigenin**				**Eriodictyol**				**Naringin**			
**Factor**	***DF***	***SS***	***MS***	***F***	***p***	***SS***	***MS***	***F***	***p***	***SS***	***MS***	***F***	***p***
Intercept	1	18.02	18.02	585.96	<0.001 *	178.46	178.46	29,811	<0.001 *	232.89	232.89	117.58	<0.001 *
*F*_1_	2	3.53	1.76	57.38	<0.001 *	85.97	42.98	7180	<0.001 *	76.44	38.22	19.30	<0.001 *
*F*_2_	2	0.03	0.02	0.52	0.594	0.02	0.01	1.52	0.221	17.29	8.65	4.37	0.014*
*F*_3_	1	0.06	0.06	2.00	0.159	0.01	0.01	1.07	0.303	46.78	46.78	23.62	<0.001 *
*F*_1_ × *F*_2_	4	0.08	0.02	0.63	0.642	0.03	0.01	1.07	0.373	22.06	5.52	2.78	0.028
*F*_1_ × *F*_3_	2	1.68	0.84	27.28	<0.001 *	6.39	3.20	533.85	<0.001 *	97.00	48.50	24.49	<0.001 *
*F*_2_ × *F*_3_	2	0.03	0.02	0.52	0.593	0.02	0.01	1.82	0.166	18.61	9.30	4.70	0.010*
*F*_1_ × *F*_2_ × *F*_3_	4	0.07	0.02	0.55	0.700	0.06	0.02	2.67	0.034	23.81	5.95	3.01	0.020*
		**Kaempherol**				**Kaempferol-7-*O*-glucoside**				**Isorhamnetin-3-*O*-glucoside**			
**Factor**	***DF***	***SS***	***MS***	***F***	***p***	***SS***	***MS***	***F***	***p***	***SS***	***MS***	***F***	***p***
Intercept	1	169.55	169.55	1133	<0.001 *	421.15	421.15	383.12	<0.001 *	3992	3992	96.69	<0.001 *
*F*_1_	2	58.51	29.25	195.44	<0.001 *	66.25	33.13	30.13	<0.001 *	1978	988.90	23.95	<0.001 *
*F*_2_	2	2.11	1.055	7.05	0.001 *	0.35	0.18	0.16	0.851	21.92	10.96	0.27	0.767
*F*_3_	1	0.09	0.09	0.63	0.430	0.25	0.25	0.23	0.632	1053	1053	25.50	<0.001 *
*F*_1_ × *F*_2_	4	4.34	1.09	7.26	<0.001 *	0.42	0.11	0.10	0.983	29.32	7.33	0.18	0.950
F_1_ × *F*_3_	2	14.66	7.33	48.97	<0.001 *	18.96	9.48	8.62	<0.001 *	2394	1197	28.99	<0.001 *
*F*_2_ × *F*_3_	2	0.17	0.09	0.58	0.560	0.31	0.15	0.14	0.870	20.37	10.18	0.25	0.782
*F*_1_ × *F*_2_ × *F*_3_	4	0.43	0.11	0.71	0.584	0.38	0.10	0.09	0.986	47.17	11.79	0.29	0.887

**Table 3:** Ultra-high performance liquid chromatography-MS^4^ (UHPLC-MS^4^) data about the identification of the main compounds in the samples.

No.	Compound Name	*t*_R_, min	Molecular Formula, [M–H]^−^	Calculated Mass, [M–H]^−^	Exact Mass, [M–H]^−^	Δ ppm	MS^2^ Fragments, (% Base Peak)	MS^3^ Fragments, (% Base Peak)	MS^4^ Fragments, (% Base Peak)
**1**	**Protocatechuic acid hexoside**	6.55	C_13_H_15_O_9_^−^	315.072156	315.07185	0.98	108 (10), 109 (11), 147(38), 152 (42), **153** (100), 163 (10), 165 (12)	108 (21), **109** (100)	**81** (100)
**2**	**Protocatechuic acid**	6.66	C_7_H_5_O_4_^−^	153.019332	153.01947	−0.89	107 (89), 108 (33), **109** (100), 110 (25), 123 (41), 125 (79), 136 (24)	65 (28), 67 (36), 81 (59), 83 (27), **91** (100)	
**3**	**Protocatechuic acid pentosylhexoside**	6.72	C_18_H_23_O_13_^−^	447.114415	447.11415	0.60	**152** (100), 153 (35), 163 (59), 177 (31), 179 (31), 271 (36), 315 (87)	**108** (100)	
**4**	**Caffeic acid hexoside 1**	6.84	C_15_H_17_O_9_^−^	341.087806	341.08804	−0.68	147 (19), 153 (25), 161 (33), **179** (100), 180 (9), 203 (10), 251 (13)	**135** (100)	
**5**	***p*** **-Coumaric acid**	6.91	C_9_H_7_O_3_^−^	163.040068	163.04010	−0.17	103 (82), 117 (68), 119 (96), 121 (61), 133 (71), **135** (100), 136 (76)	75 (24), **107** (100), 108 (12)	
**6**	**Caffeic acid hexoside 2**	7.03	C_15_H_17_O_9_^−^	341.087806	341.08754	0.77	147 (19), 153 (25), 161 (33), **179** (100), 180 (9), 203 (10), 251 (13)	**135** (100)	
**7**	**Caffeic acid hexoside 3**	7.35	C_15_H_17_O_9_^−^	341.087806	341.08769	0.33	135 (9), 147 (42), 153 (26), 161 (37), **179** (100), 180 (10), 203 (7)	**135** (100)	79 (40), **106** (100), 107 (52)
**8**	**5-*O*-Caffeoylquinic acid**	7.47	C_16_H_17_O_9_^−^	353.087806	353.08760	0.58	179 (3), **191** (100)	**85** (100), 93 (60), 111 (36), 127 (90), 171 (25), 173 (61)	**57** (100)
**9**	***p*** **-Coumaric acid hexoside**	7.84	C_15_H_17_O_8_^−^	325.092891	325.09274	0.48	119 (11), **145** (100), 146 (10), 163 (87), 187 (40), 265 (16), 289 (58)	**117** (100), 127 (3)	
**10**	**5-*O*-Caffeoylquinic acid isomer**	7.90	C_16_H_17_O_9_^−^	353.087806	353.08766	0.41	**191** (100), 192 (3)	**85** (100), 93 (60), 111 (34), 127 (83), 171 (24), 173 (57)	**57** (100)
**11**	**Methyl-3-*O*-caffeoylquinate**	8.03	C_17_H_19_O_9_^−^	367.103456	367.10338	0.22	135 (46), **161** (100), 162 (10), 179 (52), 320 (12), 321 (13), 329 (74)	**133** (100)	
**12**	**3-*p*-Coumaroylquinic acid**	8.10	C_16_H_17_O_8_^−^	337.092891	337.09273	0.48	163 (5), **173** (100)	59 (7), 71 (20), **93** (100), 109 (7), 111 (49), 155 (11)	
**13**	**Methyl-5-*O*-caffeoylquinate**	8.45	C_17_H_19_O_9_^−^	367.103456	367.10310	0.96	134 (3), 135 (47), 136 (4), 161 (12), **179** (100), 180 (9), 191 (22)	**135** (100)	89 (38), 117 (13), **135** (100)
**14**	**Caffeic acid**	8.54	C_9_H_7_O_4_^−^	179.034982	179.03507	−0.49	**135** (100)	**107** (100)	
**15**	**Methyl-3-*p*-coumaroylquinate**	8.73	C_17_H_19_O_8_^−^	351.108541	351.10846	0.24	117 (5), 119 (9), **145** (100), 146 (5)	**117** (100), 145 (3)	
**16**	**Methyl-5-*O*-caffeoylquinate isomer**	8.87	C_17_H_19_O_9_^−^	367.103456	367.10332	0.37	134 (3), 135 (47), 136 (4), 161 (10), **179** (100), 180 (8), 191 (20)	**135** (100)	91 (68), 106 (29), 107 (81), **135** (100)
**17**	**Methyl-5-*p*-coumaroylquinate**	8.93	C_17_H_19_O_8_^−^	351.108541	351.10856	−0.06	119 (17), 145 (10), **163** (100), 164 (7)	**119** (100)	**93** (100)
**18**	**Methyl-5-*p*-coumaroylquinate isomer**	9.38	C_17_H_19_O_8_^−^	351.108541	351.10865	−0.31	119 (19), 145 (5), **163** (100), 164 (7)	**119** (100)	**93** (100), 119 (9), 135 (72)
**19**	**Rosmarinic acid**	9.65	C_18_H_15_O_8_^−^	359.077241	359.07707	0.47	133 (5), **161** (100), 162 (9), 179 (22), 197 (20), 223 (7), 313 (4)	**133** (100)	
**20**	**Caffeoyl-feruloylquinic acid**	10.22	C_26_H_25_O_12_^−^	529.135150	529.13492	0.43	161 (8), 179 (6), 349 (8), **367** (100), 368 (18)	134 (5), 35 (60), 161 (76), **179** (100), 191 (20), 193 (5)	**135** (100)
**21**	**Prodelphinidin B type ((epi)gallocatechin-(epi)catechin)**	6.39	C_30_H_25_O_13_^−^	593.130065	593.12993	0.22	289 (5), 315 (11), **441** (100), 442 (19)	153 (34), 161 (9), 271 (14), 287 (14), 289 (50), **315** (100)	151 (5), **153** (100), 161 (26), 193 (3), 297 (21)
**22**	**(Epi)catechin-hexoside**	6.99	C_21_H_23_O_11_^−^	451.124585	451.12435	0.52	245 (12), **289** (100), 290 (16), 405 (3)	179 (8), 203 (13), 205 (38), 231 (6), **245** (100), 247 (7)	161 (20), 175 (17), 187 (33), 188 (20), **203** (100)
**23**	**Procyanidin B type 1 (catechin-(epi)catechin)**	7.08	C_30_H_25_O_12_^−^	577.135150	577.13502	0.23	289 (24), 407 (61), 408 (13), **425** (100), 426 (16), 451 (26), 559 (8)	273 (7), 381 (4), **407** (100)	281 (93), 283 (34), **285** (100), 297 (35), 389 (31)
**24**	**Epiafzelechin-3-*O*-gallate**	7.67	C_22_H_17_O_9_^−^	425.087806	425.08756	0.58	243 (21), 273 (11), 285 (6), 379 (7), 381 (10), **407** (100), 408 (33)	256 (27), 281 (65), 283 (30), **285** (100), 297 (28), 389 (21)	213 (19), 241 (4), 242 (8), **257** (100), 258 (13)
**25**	**Procyanidin B type 2 (catechin-(epi)catechin)**	7.67	C_30_H_25_O_12_^−^	577.135150	577.13510	0.09	287 (6), 289 (17), 407 (51), 408 (10), **425** (100), 426 (11), 451 (18)	273 (7), 381 (4), **407** (100)	281 (87), 283 (30), **285** (100), 297 (27), 389 (30)
**26**	**Catechin**	8.00	C_15_H_13_O_6_^−^	289.071762	289.07159	0.61	179 (12), 203 (10), 205 (38), 231 (6), **245** (100), 246 (10), 247 (6)	161 (19), 175 (10), 187 (23), 188 (14), **203** (100), 227 (26)	157 (10), 161 (33), **175** (100), 185 (16), 188 (48)
**27**	**Procyanidin B type 3 (catechin-(epi)catechin)**	8.42	C_30_H_25_O_12_^−^	577.135150	577.13564	−0.85	287 (11), 289 (16), 407 (45), 408 (9), **425** (100), 426 (14), 451 (19)	273 (9), 381 (6), **407** (100)	**281** (100), 283 (34), 285 (84), 297 (29), 389 (34)
**28**	**Epicatechin**	8.55	C_15_H_13_O_6_^−^	289.071762	289.07176	0.00	125 (19), 167 (35), 203 (8), 205 (30), **245** (100), 246 (9), 271 (18)	161 (23), 185 (37), 187 (17), 201 (19), **203** (100), 227 (67)	
**29**	**(Epi)catechin-methyl(epi)gallocatechin**	8.79	C_31_H_27_O_13_^−^	607.145715	607.14571	0.01	287 (79), 405 (46), 423 (33), 437 (37), 449 (22), 455 (95), **575** (100)	245 (8), **287** (100), 405 (22), 413 (20), 423 (35), 449 (48)	**125** (100), 161 (5), 243 (12), 245 (5)
**31**	**Quercetin-3-*O*-rhamnoside**	9.23	C_21_H_19_O_11_^−^	447.093285	447.09339	−0.23	285 (3), 299 (4), 300 (28), **301** (100), 302 (11)	151 (82), **179** (100), 257 (11), 72 (10), 273 (19), 283 (20)	**151** (100)
**32**	**Kaempferol-3-*O*-pentoside**	9.56	C_20_H_17_O_10_^−^	417.082720	417.08261	0.26	255 (5), 283 (3), 284 (66), **285** (100), 286 (14), 327 (4)	163 (20), 229 (45), 241 (29), 256 (46), **257** (100), 267 (42)	163 (85), 212 (14), 213 (22), **229** (100), 239 (56)
**33**	**Kaempferol-3-*O*-rhamnoside**	9.73	C_21_H_19_O_10_^−^	431.098371	431.09814	0.55	255 (6), 283 (7), 284 (40), **285** (100), 286 (16), 327 (5)	213 (28), 229 (41), 241 (36), 256 (70), **257** (100), 267 (43)	163 (63), 213 (19), 227 (14), **229** (100), 239 (22)
**34**	**Phloretin 2′-*O*-(2′′-*O*-pentosylhexoside)**	9.00	C_26_H_31_O_14_^−^	567.171929	567.17197	−0.07	167 (7), **273** (100), 274 (14)	123 (4), 125 (4), **167** (100)	**123** (100), 125 (13), 151 (3)
**35**	**Phloretin**	9.58	C_15_H_13_O_5_^−^	273.076847	273.07672	0.46	123 (4), 125 (4), **167** (100), 168 (7)	**123** (100), 125 (13), 151 (3)	67 (3), **81** (100), 95 (55), 108 (3)
**36**	**Phloretin-2′-O-hexoside (Phlorizin)**	9.59	C_21_H_23_O_10_^−^	435.129671	435.12950	0.39	**273** (100), 274 (13)	123 (5), 125 (3), **167** (100)	**123** (100), 125 (14), 151 (3)

**Table 4:** Collected samples and their cultivar type.

Number	Sort	Peel Color	Cultivar Type
1.	Red Delicious	Red	Standard
2.	Granny Smith	Green	Standard
3.	Idared	Yellow—red	Standard
4.	Golden Delicious	Yellow	Standard
5.	Jonagold	Yellow—red	Standard
6.	Prima	Red—yellow	Resistant
7.	Gala Galax	Yellow—red	Resistant
8.	William’s Pride	Red	Resistant
9.	Rewena	Red—yellow	Resistant
10.	Topaz	Yellow—red	Resistant
11.	Remura	Red—yellow	Resistant
12.	Zaječarska duguljasta	Red	Autochthonous
13.	MioničkaTikvara	Yellow—red	Autochthonous
14.	Zaječarski delišes	Red—yellow	Autochthonous
15.	Gružanjaska letnja kolačara	Red	Autochthonous
16.	Sećeruša	Red	Autochthonous
17.	Pamuklija	Yellow—red	Autochthonous
18.	Demirka	Red—yellow	Autochthonous
19.	Jesenji jablan	Yellow	Autochthonous
20.	Kadumana	Red	Autochthonous
21.	Buzlija	Yellow—red	Autochthonous
22.	Krtajka	Red	Autochthonous
23.	Hajdučica	Red	Autochthonous
24.	Vrtiglavska slatkača	Yellow	Autochthonous
25.	Kopaoničanka	Red—yellow	Autochthonous
26.	Bela kalaćuša	Pale yellow—red	Autochthonous
27.	Loznička tikvara	Yellow—red	Autochthonous
28.	Šipura	Red—yellow	Autochthonous
29.	Šipina	Pale red—yellow	Autochthonous
30.	Kožara	Yellow—green	Autochthonous
31.	Budimka	Pale yellow—green	Autochthonous

## Data Availability

Not applicable.

## Supplementary Materials

The following supporting information can be downloaded at: https://www.mdpi.com/article/10.3390/molecules27217651/s1, Supplementary Material (separate word document) enclosing Table S1. Total phenolic content (TPC), DPPH radical scavenging activity (RSA), and individual phenolics content in the samples.; Table S2 Comparison of TPC values in the different cultivar groups and years; Table S3. Comparison of the DPPH radical inhibition values in the different cultivar groups and years.; Table S4. Univariate test for the significance of the factor effects influencing the total phenolic content (TPC) and DPPH radical scavenging activity (RSA) in the analyzed samples.; Table S5. Overview of the detected compounds by OrbiTrap in the analyzed mesocarp samples.
