# Supplementary material for: Phenolic Compounds as Phytochemical Tracers of Varietal Origin of Some Autochthonous Apple Cultivars Grown in Serbia
Source: Molecules. 2022 Nov 7;27(21):7651. doi: 10.3390/molecules27217651 (PMC9653822; doi:10.3390/molecules27217651)
Supplement: Supplementary file 1 [file molecules-27-07651-s001.zip › molecules-1986491-supplementary.pdf]

Supplementary material

Article

Phenolic Compounds as Phytochemical Tracers of Varietal Origin of Some Autochthonous Apple Cultivars Grown in Serbia

Nikola Horvacki [1], Filip Andrić [2], Uroš Gašić [3], Dejan Đurović [4], Živoslav Tešić [2], Milica Fotirić Akšić [4] and Dušanka Milojković-Opsenica [2,*]

[1] Innovation Center of the Faculty of Chemistry Ltd., Studentski trg 12-16, 11000 Belgrade, Serbia
[2] Faculty of Chemistry, University of Belgrade, P.O. Box 51, 11158 Belgrade, Serbia
[3] National Institute of Republic of Serbia, Institute for Biological Research "Siniša Stanković", University of Belgrade, 11060 Belgrade, Serbia
[4] Faculty of Agriculture, University of Belgrade, Nemanjina 6, 11080 Belgrade, Serbia
* Correspondence: dusankam@chem.bg.ac.rs

Content

Table S1. Total phenolic content (TPC), DPPH radical scavenging activity (RSA) and individual phenolics content in the samples.
Table S2. Comparison of TPC values in different cultivar groups and years.
Table S3. Comparison of DPPH radical inhibition values in different cultivar groups and years.
Table S4. Univariate test for significance of factor effects influencing total phenolic content (TPC) and DPPH radical scavenging activity (RSA) in the analysed samples.
Table S5. Overview of detected compounds by OrbiTrap in the analyzed mesocarp samples.

Table S1. Total phenolic content (TPC), DPPH radical scavenging activity (RSA) and individual phenolics content in the samples.

Tissue	Mesocarp														
**Year**	2018														
**Cultivar number[1]**	1	2	3	4	5	6	7	8	9	10	11	12	13	14	15
**Type[2]**	S	S	S	S	S	R	R	R	R	R	R	A	A	A	A
**TPC[3]**	0.49	1.41	0.67	0.62	0.46	0.92	0.39	0.27	0.33	0.64	0.53	2.44	0.82	1.03	4.51
**RSA[4]**	17.58	20.97	18.63	18.71	8.94	19.70	15.86	7.98	8.57	16.78	19.73	30.30	16.03	17.84	45.52
**5-*O*-Caffeoylquinic acid[5]**	17.88	36.53	68.37	58.27	17.34	134.03	37.81	30.34	28.93	29.59	6.73	128.16	101.23	37.20	206.75
**Caffeic acid**	0.06	0.13	0.27	0.22	0.07	0.49	0.14	0.13	0.10	0.09	0.04	0.39	0.43	0.14	0.84
**Phloretin**	0.05	0.06	0.05	0.05	0.05	0.06	0.05	0.05	0.05	0.05	0.04	0.07	0.09	0.09	0.06
**Phlorizin**	1.24	2.52	2.05	2.87	0.46	3.82	0.83	0.78	0.57	0.75	0.34	2.50	4.28	3.32	4.94
**Quercetin**	0.10	0.11	0.13	0.21	0.11	0.25	0.12	0.08	0.06	0.03	0.11	0.29	0.44	0.26	0.24
**Rutin**	0.00	0.00	0.00	0.02	0.03	0.06	0.04	0.00	0.02	0.00	0.03	0.00	0.10	0.00	0.29
**Quercetin-3-*O*-glucoside**	0.32	0.29	0.93	0.70	0.28	1.84	1.34	0.87	0.12	0.06	1.35	0.53	1.37	0.51	1.83
**Quercetin-3-*O*-rhamnoside**	0.63	1.62	0.48	2.72	2.67	4.09	1.03	1.77	1.64	0.45	0.51	3.18	0.60	0.79	2.13
**Isorhamnetin-3-*O*-glucoside**	1.63	0.08	0.04	0.06	0.02	0.05	0.02	0.02	1.68	1.67	1.69	1.85	1.83	1.81	1.84
**Kaempferol**	0.53	0.54	0.56	0.54	0.55	0.55	0.55	0.55	0.55	0.55	0.55	0.55	0.55	0.55	0.54
**Naringenin**	0.54	0.55	0.57	0.55	0.56	0.56	0.56	0.56	0.56	0.56	0.56	0.56	0.56	0.56	0.55
**Protocatechuic acid**	0.58	0.59	0.60	0.59	0.60	0.60	0.60	0.59	0.59	0.59	0.60	0.59	0.60	0.60	0.59
***p*-Hydroxybenzoic acid**	0.38	0.39	0.40	0.38	0.39	0.39	0.39	0.39	0.39	0.39	0.39	0.39	0.40	0.39	0.39
**Catechin**	6.37	35.67	6.04	8.84	2.68	14.80	4.84	0.70	3.33	7.11	6.14	40.79	7.63	11.69	14.90
***p*-Coumaric acid**	0.01	0.05	0.07	0.02	0.00	0.00	0.02	0.00	0.00	0.00	0.03	0.04	0.07	0.04	0.21
**Isorhamnetin-3-*O*-rutinoside**	0.45	0.46	0.47	0.46	0.47	0.47	0.47	0.47	0.47	0.47	0.47	0.47	0.47	0.47	0.46
**Ferulic acid**	0.65	0.01	0.68	0.67	0.68	0.02	0.68	0.02	0.02	0.67	0.02	0.04	0.02	0.02	0.04
**Kaempferol-7-*O*-glucoside**	0.56	0.45	0.28	0.26	0.27	0.52	0.19	0.83	0.20	0.16	0.32	0.53	0.33	0.77	0.52
**Apigenin**	0.39	0.40	0.41	0.40	0.41	0.41	0.41	0.40	0.40	0.40	0.41	0.40	0.41	0.41	0.40
**Luteolin**	0.32	0.33	0.34	0.33	0.33	0.33	0.33	0.33	0.33	0.33	0.33	0.33	0.34	0.33	0.33
**Eriodictyol**	0.58	0.59	0.61	0.59	0.60	0.60	0.60	0.60	0.60	0.60	0.60	0.60	0.61	0.60	0.59
**Gallic acid**	0.47	0.48	0.49	0.48	0.48	0.49	0.48	0.48	0.48	0.48	0.49	0.48	0.49	0.49	0.48
**Naringin**	0.36	0.37	0.38	0.37	0.38	0.38	0.38	0.37	0.37	0.37	0.38	0.37	0.38	0.38	0.37

1 – refer to the table 4. in article (as a list of collected cultivars).

2 – S- standard, R- resistant, A- autocthtonous type of cultivar.

3 – expressed in g/kg equavilents gallic acid.

4 – expressed in mmol/kg trollox equavilents.

5 – expressed in mg/kg (all individual compounds presented in the table).

Table S1. Continued

Tissue	Mesocarp														
**Year**	2018														
**Cultivar number[1]**	16	17	18	19	20	21	22	23	24	25	26	27	28	29	30
**Type[2]**	A	A	A	A	A	A	A	A	A	A	A	A	A	A	A
**TPC[3]**	0.83	1.43	1.34	2.15	1.31	1.23	1.23	0.99	1.77	4.42	1.67	1.30	0.74	1.50	1.38
**RSA[4]**	10.25	18.82	21.20	18.59	26.78	19.26	17.20	17.23	8.48	19.02	16.55	16.53	18.46	20.41	17.21
**5-*O*-Caffeoylquinic acid[5]**	34.88	196.92	163.34	130.34	191.04	170.71	187.21	82.67	103.51	212.18	185.19	132.68	135.58	144.25	148.55
**Caffeic acid**	0.14	0.75	0.65	0.49	0.67	0.67	0.63	0.31	0.37	0.72	0.67	0.49	0.46	0.51	0.48
**Phloretin**	0.05	0.14	0.11	0.12	0.08	0.09	0.07	0.06	0.07	0.11	0.06	0.10	0.06	0.07	0.05
**Phlorizin**	1.76	8.42	6.22	9.00	5.17	5.44	4.10	2.26	4.97	7.99	2.69	4.19	2.26	3.91	2.68
**Quercetin**	0.16	0.74	0.36	0.64	0.64	0.15	0.10	0.26	0.08	0.08	0.14	0.13	0.03	0.45	0.30
**Rutin**	0.00	0.60	0.04	0.02	0.00	0.01	0.00	0.03	0.05	0.31	0.02	0.00	0.00	0.16	0.04
**Quercetin-3-*O*-glucoside**	0.56	4.71	0.63	1.14	1.39	0.60	0.23	0.82	0.63	0.80	0.48	0.49	0.50	5.45	0.47
**Quercetin-3-*O*-rhamnoside**	0.97	1.15	0.38	5.19	2.74	0.19	1.39	3.20	0.31	0.80	1.47	0.17	0.78	3.75	1.61
**Isorhamnetin-3-*O*-glucoside**	0.03	1.65	1.69	0.03	0.12	1.71	0.04	0.01	1.56	1.66	1.70	0.02	0.02	0.02	1.68
**Kaempferol**	0.56	0.54	0.55	0.56	0.55	0.56	0.56	0.55	0.51	0.54	0.55	0.52	0.55	0.56	0.55
**Naringenin**	0.57	0.55	0.56	0.57	0.56	0.57	0.57	0.56	0.52	0.55	0.56	0.53	0.56	0.57	0.56
**Protocatechuic acid**	0.60	0.59	0.60	0.61	0.60	0.61	0.61	0.60	0.55	0.59	0.60	0.57	0.60	0.60	0.60
***p*-Hydroxybenzoic acid**	0.40	0.38	0.39	0.40	0.39	0.40	0.40	0.39	0.36	0.39	0.39	0.37	0.39	0.40	0.39
**Catechin**	7.23	13.37	18.63	53.08	9.95	15.39	10.42	19.05	4.37	12.25	29.97	12.09	3.91	27.79	18.90
***p*-Coumaric acid**	0.03	0.10	0.11	0.35	0.15	0.07	0.09	0.09	0.06	0.08	0.03	0.07	0.08	0.04	0.05
**Isorhamnetin-3-*O*-rutinoside**	0.48	0.46	0.47	0.48	0.47	0.48	0.48	0.47	0.43	0.46	0.47	0.45	0.47	0.47	0.47
**Ferulic acid**	0.69	0.04	0.03	0.12	0.07	0.69	0.02	0.19	0.02	0.03	0.01	0.02	0.02	0.05	0.03
**Kaempferol-7-*O*-glucoside**	0.87	0.54	0.23	1.16	0.43	0.25	0.39	0.32	0.21	0.50	0.45	0.22	0.65	0.58	0.31
**Apigenin**	0.41	0.40	0.41	0.41	0.41	0.41	0.41	0.41	0.38	0.40	0.41	0.39	0.41	0.41	0.41
**Luteolin**	0.34	0.33	0.34	0.34	0.33	0.34	0.34	0.33	0.31	0.33	0.34	0.32	0.34	0.34	0.33
**Eriodictyol**	0.61	0.59	0.60	0.61	0.60	0.61	0.61	0.60	0.56	0.59	0.61	0.57	0.60	0.61	0.60
**Gallic acid**	0.49	0.48	0.49	0.49	0.49	0.49	0.49	0.49	0.45	0.48	0.49	0.46	0.49	0.49	0.48
**Naringin**	0.38	0.37	0.38	0.38	0.38	0.38	0.38	0.38	0.35	0.37	0.38	0.36	0.38	0.38	0.38

1 – refer to the table 4. in article (as a list of collected cultivars).

2 – S- standard, R- resistant, A- autocthtonous type of cultivar.

3 – expressed in g/kg equavilents gallic acid.

4 – expressed in mmol/kg trollox equavilents.

5 – expressed in mg/kg (all individual compounds presented in the table).

Table S1. Continued

**Tissue**	Mesocarp														
**Year**	2019														
**Cultivar number[1]**	1	2	3	4	5	6	7	8	9	10	11	12	13	14	15
**Type[2]**	S	S	S	S	S	R	R	R	R	R	R	A	A	A	A
**TPC[3]**	0.51	0.73	0.59	0.42	0.22	0.11	0.12	0.14	1.61	0.70	0.31	1.96	2.59	0.88	2.15
**RSA[4]**	9.28	9.44	9.02	7.79	9.64	9.58	7.75	8.04	8.79	8.28	7.91	9.31	9.17	9.37	10.17
**5-*O*-Caffeoylquinic acid[5]**	1.86	3.23	6.46	4.05	5.54	3.55	2.77	10.72	5.91	8.43	0.48	18.82	15.66	7.22	41.99
**Caffeic acid**	1.02	1.02	1.01	0.99	1.04	1.00	0.97	0.95	1.01	0.99	0.93	0.91	1.01	1.00	0.98
**Phloretin**	0.04	0.05	0.06	0.06	0.04	0.06	0.04	0.09	0.05	0.04	0.04	0.07	0.17	0.07	0.15
**Phlorizin**	0.87	0.62	0.91	0.96	0.55	0.39	0.22	1.14	0.55	0.43	0.11	1.06	2.40	2.28	4.40
**Quercetin**	1.76	1.75	1.74	1.70	1.79	1.73	1.67	1.63	1.74	1.71	1.60	1.57	1.73	1.72	1.69
**Rutin**	0.03	0.05	0.07	0.08	0.07	0.03	0.04	0.04	0.07	0.04	0.03	0.05	0.10	0.04	0.14
**Quercetin-3-*O*-glucoside**	0.19	0.22	0.36	0.37	0.94	0.17	0.18	0.24	0.24	0.23	0.18	0.36	0.53	0.23	0.37
**Quercetin-3-*O*-rhamnoside**	0.28	0.45	0.17	1.14	1.02	0.26	0.26	0.29	0.68	0.18	0.13	1.04	0.10	0.29	0.12
**Isorhamnetin-3-*O*-glucoside**	0.02	0.02	0.01	0.08	0.05	0.01	0.02	0.01	0.01	0.00	0.01	0.06	0.10	0.07	0.04
**Kaempferol**	1.07	1.06	1.05	1.03	1.08	1.04	1.01	0.99	1.05	1.03	0.97	0.95	1.05	1.04	1.02
**Naringenin**	1.09	1.08	1.07	1.05	1.10	1.06	1.03	1.01	1.07	1.05	0.98	0.96	1.07	1.06	1.04
**Protocatechuic acid**	1.16	1.15	1.14	1.12	1.18	1.13	1.10	1.07	1.14	1.12	1.05	1.03	1.14	1.13	1.11
***p*-Hydroxybenzoic acid**	0.76	0.76	0.75	0.73	0.77	0.74	0.72	0.70	0.75	0.74	0.69	0.67	0.75	0.74	0.73
**Catechin**	1.40	1.39	1.38	1.35	1.42	1.37	1.33	1.30	1.38	1.36	1.27	1.25	1.38	1.37	1.34
***p*-Coumaric acid**	0.34	0.34	0.33	0.33	0.34	0.33	0.32	0.31	0.33	0.33	0.31	0.30	0.33	0.33	0.32
**Isorhamnetin-3-*O*-rutinoside**	0.91	0.91	0.90	0.88	0.92	0.89	0.86	0.84	0.90	0.88	0.83	0.81	0.90	0.89	0.87
**Ferulic acid**	1.31	1.31	1.29	1.27	1.33	1.29	1.25	1.22	1.30	1.27	1.19	1.17	1.29	1.29	1.26
**Kaempferol-7-*O*-glucoside**	1.26	1.25	1.24	1.21	1.28	1.23	1.19	1.17	1.24	1.22	1.14	1.12	1.24	1.23	1.20
**Apigenin**	0.79	0.78	0.78	0.76	0.80	0.77	0.75	0.73	0.78	0.76	0.72	0.70	0.78	0.77	0.75
**Luteolin**	0.65	0.64	0.64	0.62	0.66	0.63	0.61	0.60	0.64	0.63	0.59	0.57	0.64	0.63	0.62
**Eriodictyol**	1.17	1.16	1.15	1.12	1.18	1.14	1.10	1.08	1.15	1.13	1.06	1.03	1.14	1.14	1.11
**Gallic acid**	0.94	0.94	0.93	0.91	0.96	0.92	0.89	0.87	0.93	0.91	0.85	0.84	0.93	0.92	0.90
**Naringin**	0.02	0.22	0.29	0.12	0.23	0.03	0.05	0.14	0.13	0.14	0.04	0.89	1.14	0.08	0.38

1 – refer to the table 4. in article (as a list of collected cultivars).

2 – S- standard, R- resistant, A- autocthtonous type of cultivar.

3 – expressed in g/kg equavilents gallic acid.

4 – expressed in mmol/kg trollox equavilents.

5 – expressed in mg/kg (all individual compounds presented in the table).

Table S1. Continued

Tissue	Mesocarp															
**Year**	2019															
**Cultivar number**	16	17	18	19	20	21	22	23	24	25	26	27	28	29	30	31[6]
**Type**	A	A	A	A	A	A	A	A	A	A	A	A	A	A	A	A
**TPC**	1.30	1.01	0.97	1.85	1.44	0.81	1.40	1.53	1.77	1.57	1.19	1.57	1.17	2.17	1.98	1.07
**RSA**	10.97	10.17	11.91	9.50	10.42	8.62	8.13	9.52	9.22	6.54	9.36	6.03	9.95	10.41	11.42	8.81
**5-*O*-Caffeoylquinic acid[5]**	10.31	14.90	7.78	16.94	20.66	11.09	30.62	21.07	30.06	25.70	17.83	23.66	28.50	22.10	24.39	17.80
**Caffeic acid**	1.04	1.00	0.98	0.97	1.02	0.99	0.93	0.94	0.97	1.01	0.97	1.00	0.95	0.97	1.03	1.01
**Phloretin**	0.20	0.17	0.08	0.23	0.09	0.11	0.12	0.09	0.21	0.24	0.10	0.22	0.08	0.15	0.26	0.10
**Phlorizin**	3.99	2.19	1.32	3.65	1.50	1.90	2.67	1.58	3.70	5.93	1.54	3.59	1.36	1.65	5.11	1.65
**Quercetin**	1.79	1.73	1.69	1.67	1.75	1.71	1.61	1.62	1.67	1.73	1.67	1.73	1.64	1.66	1.77	1.74
**Rutin**	0.06	0.05	0.05	0.11	0.03	0.05	0.05	0.06	0.12	0.03	0.23	0.08	0.05	0.04	0.06	0.11
**Quercetin-3-*O*-glucoside**	0.37	0.37	0.58	1.02	0.31	0.40	0.49	0.60	0.61	0.21	1.01	0.70	0.30	0.22	0.34	0.56
**Quercetin-3-*O*-rhamnoside**	0.36	0.08	0.68	1.04	0.67	0.05	0.57	0.63	0.10	0.13	0.55	0.07	0.31	0.23	0.66	0.34
**Isorhamnetin-3-*O*-glucoside**	0.06	0.05	0.04	0.08	0.03	0.06	0.04	0.06	0.04	0.05	0.08	0.04	0.02	0.00	0.03	0.04
**Kaempferol**	1.08	1.04	1.02	1.01	1.06	1.03	0.97	0.98	1.01	1.05	1.01	1.04	0.99	1.01	1.07	1.05
**Naringenin**	1.10	1.06	1.04	1.03	1.08	1.05	0.99	1.00	1.03	1.07	1.03	1.06	1.01	1.02	1.09	1.07
**Protocatechuic acid**	1.18	1.13	1.11	1.10	1.15	1.12	1.06	1.06	1.09	1.14	1.10	1.13	1.07	1.09	1.16	1.14
***p*-Hydroxybenzoic acid**	0.77	0.74	0.73	0.72	0.75	0.74	0.69	0.70	0.72	0.75	0.72	0.74	0.70	0.72	0.76	0.75
**Catechin**	1.43	1.37	1.34	1.33	1.39	1.36	1.28	1.29	1.32	1.38	1.33	1.37	1.30	1.32	1.40	1.39
***p*-Coumaric acid**	0.34	0.33	0.32	0.32	0.34	0.33	0.31	0.31	0.32	0.33	0.32	0.33	0.31	0.32	0.34	0.33
**Isorhamnetin-3-*O*-rutinoside**	0.93	0.89	0.87	0.86	0.90	0.88	0.83	0.84	0.86	0.89	0.86	0.89	0.85	0.86	0.91	0.90
**Ferulic acid**	1.34	1.29	1.26	1.25	1.31	1.27	1.20	1.21	1.24	1.29	1.24	1.29	1.22	1.24	1.32	1.30
**Kaempferol-7-*O*-glucoside**	1.28	1.23	1.20	1.19	1.25	1.22	1.15	1.15	1.19	1.24	1.19	1.23	1.17	1.19	1.26	1.24
**Apigenin**	0.80	0.77	0.76	0.75	0.78	0.76	0.72	0.72	0.75	0.77	0.75	0.77	0.73	0.74	0.79	0.78
**Luteolin**	0.66	0.63	0.62	0.61	0.64	0.63	0.59	0.59	0.61	0.64	0.61	0.63	0.60	0.61	0.65	0.64
**Eriodictyol**	1.18	1.14	1.12	1.10	1.16	1.13	1.06	1.07	1.10	1.14	1.10	1.14	1.08	1.10	1.17	1.15
**Gallic acid**	0.96	0.92	0.90	0.89	0.93	0.91	0.86	0.86	0.89	0.92	0.89	0.92	0.87	0.89	0.94	0.93
**Naringin**	0.16	1.02	0.20	0.88	0.87	0.80	0.88	0.64	1.86	0.49	0.24	1.57	0.61	0.60	1.53	0.17

1 – refer to the table 4. in article (as a list of collected cultivars).

2 – S- standard, R- resistant, A- autocthtonous type of cultivar.

3 – expressed in g/kg equavilents gallic acid.

4 – expressed in mmol/kg trollox equavilents.

5 – expressed in mg/kg (all individual compounds presented in the table).

6 – sample unavvaible in 2018.

Table S1. Continued

Tissue	Peel														
**Year**	2018														
**Cultivar number**[1]	1	2	3	4	5	6	7	8	9	10	11	12	13	14	15
**Type**[2]	S	S	S	S	S	R	R	R	R	R	R	A	A	A	A
**TPC**[3]	3.67	5.31	6.51	3.43	4.55	5.44	4.52	3.55	3.30	3.13	3.50	7.71	6.78	7.52	6.70
**RSA**[4]	50.75	57.64	42.08	29.34	36.35	61.21	33.25	30.81	54.82	26.28	31.12	49.35	40.93	43.19	41.46
**5-*O*-Caffeoylquinic acid**[5]	4.55	7.58	85.52	63.33	12.64	206.53	49.84	11.99	25.73	12.46	3.81	80.99	312.00	24.77	557.46
**Caffeic acid**	0.03	0.05	0.34	0.26	0.08	0.83	0.24	0.06	0.13	0.07	0.05	0.33	1.30	0.15	1.69
**Phloretin**	0.14	0.07	0.16	0.17	0.20	0.17	0.14	0.07	0.05	0.11	0.14	0.37	1.25	0.61	1.00
**Phlorizin**	20.66	6.10	23.09	19.18	10.52	13.77	12.14	3.95	4.03	4.05	4.99	31.99	42.85	38.31	30.21
**Quercetin**	28.22	21.00	30.59	27.83	28.62	33.74	25.60	20.44	9.53	19.42	15.91	31.67	33.07	45.77	20.18
**Rutin**	2.20	13.41	3.26	5.01	1.87	5.59	3.12	3.67	3.24	5.70	1.28	2.55	9.41	4.43	11.96
**Quercetin-3-*O*-glucoside**	49.12	62.71	57.45	53.31	34.87	55.39	48.39	45.36	35.21	41.72	24.24	45.43	45.11	54.00	32.31
**Quercetin-3-*O*-rhamnoside**	25.63	38.94	27.15	26.00	29.40	67.68	23.90	48.94	47.49	42.03	6.71	22.66	16.99	24.11	17.79
**Isorhamnetin-3-*O*-glucoside**	0.65	0.66	3.27	0.21	1.03	2.24	0.28	1.47	0.06	0.14	0.04	4.55	0.08	1.51	0.08
**Kaempferol**	0.23	0.15	0.12	0.24	0.23	0.72	0.19	0.30	0.15	0.35	0.14	0.23	0.42	0.67	0.41
**Naringenin**	0.06	0.04	0.08	0.06	0.12	0.08	0.06	0.09	0.02	0.04	0.57	0.14	0.08	0.56	0.06
**Protocatechuic acid**	0.51	0.51	0.86	0.52	0.57	1.01	0.68	0.92	0.57	0.54	0.97	0.57	0.65	0.71	0.99
***p*-Hydroxybenzoic acid**	0.07	0.11	0.10	0.07	0.20	0.05	0.08	0.15	0.08	0.15	0.13	0.09	0.12	0.16	0.30
**Catechin**	28.26	33.26	41.53	24.10	21.31	35.51	43.72	6.79	15.69	16.18	21.42	74.61	59.42	50.27	30.25
***p*-Coumaric acid**	0.04	0.05	0.09	0.05	0.06	0.17	0.07	0.05	0.05	0.07	0.08	0.05	0.34	0.06	0.69
**Isorhamnetin-3-*O*-rutinoside**	0.22	0.15	0.39	0.07	0.28	0.94	0.10	0.50	1.10	0.13	0.02	0.65	0.47	0.22	0.48
**Ferulic acid**	0.69	0.05	0.03	0.67	0.02	0.05	0.05	0.06	0.03	0.03	0.05	0.03	0.21	0.03	0.25
**Kaempferol-7-*O*-glucoside**	6.06	2.73	0.66	3.27	0.66	0.65	2.28	0.65	3.22	5.53	1.97	0.66	2.13	0.64	4.66
**Apigenin**	0.41	0.40	0.41	0.40	0.41	0.41	0.41	0.41	0.95	0.41	0.42	0.41	0.41	0.40	0.41
**Luteolin**	0.34	0.33	0.34	0.33	0.34	0.33	0.34	0.33	0.78	0.33	0.34	0.34	0.34	0.33	0.34
**Eriodictyol**	0.61	0.59	0.61	0.59	0.61	0.60	0.61	0.60	1.40	0.60	0.62	0.61	0.60	0.60	0.61
**Gallic acid**	0.49	0.48	0.49	0.48	0.49	0.48	0.49	0.49	1.13	0.49	0.50	0.49	0.49	0.48	0.49
**Naringin**	0.38	0.37	0.38	0.37	0.38	0.38	0.38	0.38	0.88	0.38	0.39	0.38	0.38	0.37	0.38

1 – refer to the table 4. in article (as a list of collected cultivars).
2 – S- standard, R- resistant, A- autocthtonous type of cultivar.
3 – expressed in g/kg equavilents gallic acid.
4 – expressed in mmol/kg trollox equavilents.
5 – expressed in mg/kg (all individual compounds presented in the table).

Table S1. Continued

Tissue	Peel														
**Year**	2018														
**Cultivar number[1]**	16	17	18	19	20	21	22	23	24	25	26	27	28	29	30
**Type[2]**	A	A	A	A	A	A	A	A	A	A	A	A	A	A	A
**TPC[3]**	6.60	6.40	5.70	8.54	7.53	7.52	4.87	4.35	5.07	8.42	7.57	7.91	5.41	8.59	9.26
**RSA[4]**	40.85	41.45	37.08	44.13	45.61	39.48	39.21	29.51	34.16	45.64	45.86	40.38	36.07	48.44	45.71
**5-*O*-Caffeoylquinic acid[5]**	36.15	428.67	430.92	93.81	503.12	214.73	224.67	96.40	326.37	269.85	257.52	227.26	235.77	272.71	312.87
**Caffeic acid**	0.12	1.52	1.64	1.05	1.71	0.80	0.89	0.49	1.35	0.84	1.19	0.79	0.78	0.81	1.29
**Phloretin**	0.68	1.26	1.21	0.92	0.38	0.48	0.18	0.21	0.48	0.35	0.12	0.77	0.27	0.53	0.15
**Phlorizin**	25.77	38.95	35.58	35.46	17.95	44.66	17.54	14.76	43.69	54.25	12.72	59.04	25.24	32.52	13.25
**Quercetin**	39.11	38.04	22.12	16.99	24.70	27.77	9.78	17.74	21.94	19.30	16.34	20.71	18.61	38.18	14.24
**Rutin**	5.98	19.70	3.87	1.71	1.26	23.24	0.45	4.30	9.92	20.62	8.12	6.52	0.67	6.29	6.09
**Quercetin-3-*O*-glucoside**	42.68	43.15	19.86	15.52	23.53	71.81	9.18	37.66	41.80	75.25	37.63	45.05	30.44	50.68	27.02
**Quercetin-3-*O*-rhamnoside**	20.46	15.32	8.50	14.00	18.80	23.20	9.09	20.87	15.38	27.26	16.00	18.84	19.12	25.26	13.00
**Isorhamnetin-3-*O*-glucoside**	2.18	0.18	0.06	0.04	1.94	0.28	2.12	0.09	0.17	0.24	0.25	0.18	2.41	0.24	0.14
**Kaempferol**	0.61	0.59	0.17	0.55	0.34	0.17	0.02	0.11	0.30	0.48	0.22	0.11	0.09	0.39	0.15
**Naringenin**	0.57	0.11	0.05	0.06	0.09	0.04	0.04	0.04	0.06	0.07	0.03	0.04	0.12	0.14	0.03
**Protocatechuic acid**	0.89	0.66	0.53	0.51	0.62	0.80	0.43	0.51	0.55	0.85	0.52	0.76	1.07	0.60	0.59
***p*-Hydroxybenzoic acid**	0.17	0.13	0.08	0.18	0.22	0.28	0.07	0.07	0.07	0.32	0.10	0.35	0.17	0.07	0.11
**Catechin**	31.55	32.30	56.05	136.11	58.32	49.52	29.85	77.83	37.78	43.97	57.66	56.56	34.24	88.20	58.57
***p*-Coumaric acid**	0.04	0.30	0.51	1.50	0.40	0.18	0.23	0.39	0.33	0.09	0.28	0.24	0.18	0.08	0.35
**Isorhamnetin-3-*O*-rutinoside**	0.65	0.48	0.47	0.48	0.31	0.46	0.26	0.05	0.47	0.49	0.12	0.13	0.21	0.47	0.07
**Ferulic acid**	0.69	0.22	0.44	0.48	0.18	0.09	0.09	0.52	0.33	0.03	0.47	0.11	0.04	0.06	0.27
**Kaempferol-7-*O*-glucoside**	12.21	3.93	1.42	1.87	0.65	3.08	0.66	1.43	2.55	7.45	2.68	2.30	0.66	3.52	2.76
**Apigenin**	0.41	0.41	0.41	0.41	0.41	0.40	0.41	0.41	0.41	0.42	0.41	0.41	0.41	0.41	0.41
**Luteolin**	0.34	0.34	0.34	0.34	0.34	0.33	0.34	0.34	0.33	0.35	0.34	0.34	0.34	0.34	0.34
**Eriodictyol**	0.61	0.61	0.61	0.61	0.61	0.59	0.61	0.61	0.60	0.62	0.61	0.61	0.61	0.61	0.61
**Gallic acid**	0.49	0.49	0.49	0.49	0.49	0.48	0.49	0.49	0.49	0.50	0.49	0.49	0.49	0.49	0.49
**Naringin**	0.38	0.38	0.38	0.38	0.38	0.37	0.38	0.38	0.38	0.39	0.38	0.38	0.38	0.38	0.38

1 – refer to the table 4. in article (as a list of collected cultivars).

2 – S- standard, R- resistant, A- autocthtonous type of cultivar.

3 – expressed in g/kg equavilents gallic acid.

4 – expressed in mmol/kg trollox equavilents.

5 – expressed in mg/kg (all individual compounds presented in the table).

Table S1. Continued

Tissue	Peel														
**Year**	2019														
**Cultivar number**	1	2	3	4	5	6	7	8	9	10	11	12	13	14	15
**Type**	S	S	S	S	S	R	R	R	R	R	R	A	A	A	A
**TPC**	21.52	24.89	16.11	9.70	37.33	11.66	7.64	15.47	18.37	20.66	10.49	22.26	25.88	22.01	23.42
**RSA**	34.60	113.34	40.95	23.56	34.36	27.32	21.84	27.21	35.45	31.07	23.42	48.58	38.99	36.24	42.92
**5-*O*-Caffeoylquinic acid**[5]	5.85	128.47	9.33	26.95	100.11	64.38	16.47	18.56	22.27	14.21	27.66	35.98	134.99	11.55	116.21
**Caffeic acid**	0.07	0.22	0.26	0.13	0.60	0.36	0.08	0.18	0.43	0.22	0.33	0.29	0.97	0.08	0.77
**Phloretin**	0.52	0.99	0.24	0.43	0.89	0.30	0.12	0.10	0.21	0.61	0.21	0.98	1.54	0.71	0.94
**Phlorizin**	17.04	42.39	7.69	10.53	30.53	9.83	2.81	1.61	4.98	12.20	6.69	21.09	45.76	23.04	36.21
**Quercetin**	57.01	20.41	37.10	32.51	27.76	20.67	14.02	41.46	44.78	92.04	18.54	70.28	30.64	51.53	55.63
**Rutin**	32.12	61.76	33.55	21.88	16.61	12.07	6.57	50.33	47.27	36.25	23.94	35.26	46.62	40.95	229.97
**Quercetin-3-*O*-glucoside**	160.14	115.36	153.66	123.16	158.20	104.07	69.73	229.18	158.33	231.86	116.11	164.90	123.63	163.25	244.14
**Quercetin-3-*O*-rhamnoside**	41.50	20.41	39.47	38.08	49.81	115.14	26.45	142.32	122.90	80.39	75.90	40.80	24.56	53.05	72.10
**Isorhamnetin-3-*O*-glucoside**	19.92	7.28	14.53	10.51	23.84	15.63	8.16	33.93	14.14	48.22	9.46	30.11	8.18	20.31	20.76
**Kaempferol**	0.87	0.56	0.85	0.63	0.60	0.80	0.51	0.84	1.45	1.32	0.68	0.74	0.60	1.09	1.56
**Naringenin**	0.21	0.33	0.14	0.13	0.19	0.11	0.05	0.15	0.08	0.24	0.10	0.29	0.28	0.45	0.19
**Protocatechuic acid**	1.49	1.39	1.40	1.45	1.49	1.50	1.47	1.45	1.49	1.50	1.42	1.50	1.51	1.49	1.49
***p*-Hydroxybenzoic acid**	0.97	0.91	0.92	0.95	0.98	0.98	0.97	0.95	0.98	0.98	0.93	0.98	0.99	0.97	0.98
**Catechin**	1.80	1.68	1.70	1.76	1.80	1.81	1.78	1.76	1.81	1.82	1.72	1.82	1.83	1.80	1.80
***p*-Coumaric acid**	0.43	0.41	0.41	0.42	0.44	0.44	0.43	0.42	0.44	0.44	0.42	0.44	0.44	0.43	0.43
**Isorhamnetin-3-*O*-rutinoside**	1.17	1.09	1.10	1.14	1.17	1.18	1.16	1.14	1.18	1.18	1.12	1.18	1.19	1.17	1.17
**Ferulic acid**	1.69	1.58	1.59	1.65	1.69	1.70	1.67	1.65	1.70	1.70	1.61	1.70	1.71	1.69	1.69
**Kaempferol-7-*O*-glucoside**	1.61	1.51	1.53	1.58	1.62	1.63	1.60	1.58	1.62	1.63	1.54	1.63	1.64	1.61	1.62
**Apigenin**	0.07	0.13	0.08	0.05	1.02	0.05	0.02	0.08	0.05	0.11	0.97	0.11	0.03	0.20	0.03
**Luteolin**	0.83	0.78	0.78	0.81	0.83	0.84	0.82	0.81	0.83	0.84	0.79	0.84	0.84	0.83	0.83
**Eriodictyol**	0.10	0.06	0.06	0.09	0.10	0.04	0.03	0.09	0.12	0.14	0.05	0.14	0.06	0.11	0.06
**Gallic acid**	1.21	1.13	1.14	1.18	1.21	1.22	1.20	1.18	1.21	1.22	1.16	1.22	1.23	1.21	1.21
**Naringin**	1.09	11.85	2.29	2.03	5.62	1.99	1.12	0.16	1.59	4.17	1.39	6.94	11.61	1.57	2.72

1 – refer to the table 4. in article.

2 – S- standard, R- resistant, A- autocthtonous.

3 – expressed in g/kg equavilents gallic acid.

4 – expressed in mmol/kg trollox equavilents.

5 – expressed in mg/kg.

Table S1. Continued

Tissue	Peel															
**Year**	2019															
**Cultivar number**	16	17	18	19	20	21	22	23	24	25	26	27	28	29	30	31[6]
**Type**	A	A	A	A	A	A	A	A	A	A	A	A	A	A	A	A
**TPC**	24.69	22.57	18.46	28.78	41.62	20.47	24.95	25.26	27.77	21.16	28.66	21.47	28.70	10.61	24.33	15.24
**RSA**	43.80	98.13	40.19	46.76	38.10	36.00	124.55	43.40	112.90	35.21	48.97	38.88	54.05	23.48	42.85	28.46
**5-*O*-Caffeoylquinic acid[5]**	15.47	60.17	16.46	24.38	67.36	94.21	67.08	50.20	128.06	89.73	121.61	127.79	94.35	1.37	82.14	74.63
**Caffeic acid**	0.07	0.51	0.34	0.30	0.46	0.35	1.49	0.24	1.04	0.85	1.16	0.93	0.32	0.08	0.71	0.46
**Phloretin**	0.84	1.40	0.47	1.51	3.51	1.22	1.14	0.91	1.57	3.26	0.36	1.67	0.84	0.20	3.88	0.33
**Phlorizin**	32.37	31.89	10.74	36.77	52.88	35.48	25.63	25.75	44.95	40.00	11.82	25.01	34.31	1.22	45.84	10.16
**Quercetin**	54.33	18.04	92.90	19.48	112.09	18.70	53.51	47.06	26.16	18.84	73.30	27.22	34.85	13.39	41.72	12.17
**Rutin**	63.71	20.06	19.36	98.76	19.62	45.39	67.03	134.38	61.36	57.98	167.03	62.38	46.11	4.30	44.22	36.04
**Quercetin-3-*O*-glucoside**	183.65	80.28	173.74	153.35	172.96	117.30	193.38	209.13	125.08	139.46	224.54	123.87	226.27	64.85	141.76	85.73
**Quercetin-3-*O*-rhamnoside**	54.16	16.13	57.65	49.84	77.79	17.31	52.86	54.38	22.08	29.94	47.81	14.37	75.22	11.12	41.29	19.66
**Isorhamnetin-3-*O*-glucoside**	22.63	4.86	18.82	11.77	44.99	6.58	74.46	17.20	7.77	11.34	19.38	7.17	36.32	7.51	13.77	7.39
**Kaempferol**	1.33	0.57	1.23	0.95	1.06	0.51	0.76	0.97	0.65	0.82	1.46	0.91	0.65	0.53	1.01	0.61
**Naringenin**	0.55	0.22	0.19	0.26	0.70	0.19	0.68	0.45	0.40	0.65	0.16	0.69	0.39	0.07	0.75	0.08
**Protocatechuic acid**	1.49	1.46	1.50	1.51	1.49	1.49	1.51	1.52	1.51	1.49	1.49	1.48	1.47	1.45	1.42	1.48
***p*-Hydroxybenzoic acid**	0.98	0.96	0.99	0.99	0.98	0.98	0.99	1.00	0.99	0.98	0.98	0.97	0.97	0.95	0.93	0.97
**Catechin**	1.81	1.77	1.82	1.83	1.80	1.81	1.82	1.84	1.83	1.81	1.81	1.79	1.78	1.75	1.72	1.79
***p*-Coumaric acid**	0.44	0.43	0.44	0.44	0.43	0.44	0.44	0.44	0.44	0.44	0.44	0.43	0.43	0.42	0.42	0.43
**Isorhamnetin-3-*O*-rutinoside**	1.18	1.15	1.18	1.19	1.17	1.17	1.18	1.20	1.19	1.17	1.17	1.17	1.16	1.14	1.12	1.17
**Ferulic acid**	1.70	1.66	1.71	1.72	1.69	1.69	1.71	1.73	1.71	1.70	1.70	1.68	1.67	1.64	1.61	1.68
**Kaempferol-7-*O*-glucoside**	1.62	1.59	1.63	1.65	1.62	1.62	1.64	1.65	1.64	1.62	1.62	1.61	1.60	1.57	1.54	1.61
**Apigenin**	1.02	0.03	0.08	0.03	0.10	0.02	0.30	1.04	0.12	1.02	0.05	0.25	0.15	0.03	0.97	1.01
**Luteolin**	0.84	0.82	0.84	0.85	0.83	0.83	0.84	0.85	0.84	0.83	0.83	0.83	0.82	0.81	0.79	0.83
**Eriodictyol**	0.11	0.03	0.14	0.11	0.22	0.05	0.23	0.09	0.09	0.12	0.11	0.11	0.08	0.05	0.30	0.04
**Gallic acid**	1.21	1.19	1.22	1.23	1.21	1.21	1.22	1.24	1.23	1.21	1.21	1.20	1.20	1.18	1.16	1.21
**Naringin**	1.81	7.48	2.33	8.33	7.42	11.37	5.13	5.64	12.48	6.29	2.90	9.13	5.14	0.22	5.70	3.15

1 – refer to the table 4. in article (as a list of collected cultivars).

2 – S- standard, R- resistant, A- autocthtonous type of cultivar.

3 – expressed in g/kg equavilents gallic acid.

4 – expressed in mmol/kg trollox equavilents.

5 – expressed in mg/kg (all individual compounds presented in the table).

6 – sample unavvaible in 2018.

Table S1. Continued

**Tissue**	Leaves															
**Year**	2018															
**Cultivar number**[1]	1	2	3	4	5	6	7	8	9	10	11	12	13	14	15	16
**Type**[2]	S	S	S	S	S	R	R	R	R	R	R	A	A	A	A	A
**TPC**[3]	67.49	81.48	64.79	88.62	80.11	75.73	74.33	64.11	64.12	74.83	78.15	73.30	82.82	61.33	61.46	17.72
**RSA**[4]	7.97	11.85	10.54	7.56	9.63	11.77	7.77	6.40	9.17	8.41	10.79	7.47	12.48	6.51	6.10	5.19
**5-*O*-Caffeoylquinic acid**[5]	69.97	87.97	77.77	46.30	63.93	102.49	69.72	101.33	74.58	79.63	60.49	71.43	178.55	116.43	105.90	23.52
**Caffeic acid**	1.62	2.55	3.19	1.59	2.38	3.08	1.87	2.37	1.87	1.68	1.46	1.98	3.95	1.03	1.41	0.30
**Phloretin**	81.67	38.85	61.83	243.70	273.32	285.53	58.16	416.08	392.33	235.46	213.48	400.51	179.21	208.41	360.40	85.56
**Phlorizin**	199.54	204.38	210.17	250.09	248.42	193.46	209.69	220.43	224.70	217.87	218.77	248.26	230.83	229.96	247.17	165.42
**Quercetin**	19.81	30.47	31.69	33.66	22.10	32.09	12.46	44.51	37.22	23.08	29.96	24.94	26.66	16.44	27.33	19.73
**Rutin**	54.80	127.08	82.18	107.59	82.98	115.36	64.08	81.14	90.62	70.31	67.29	67.40	155.58	62.05	146.33	58.48
**Quercetin-3-*O*-glucoside**	305.18	369.76	371.53	374.11	354.41	334.67	319.32	383.49	331.67	320.93	295.91	317.13	386.58	327.65	338.94	197.56
**Quercetin-3-*O*-rhamnoside**	177.11	254.60	216.40	246.63	216.21	254.79	194.56	247.87	312.50	294.45	197.15	123.18	222.76	158.18	202.10	105.73
**Isorhamnetin-3-*O*-glucoside**	6.48	6.60	6.42	6.25	6.61	6.07	6.66	6.71	6.68	6.22	6.39	6.38	6.63	6.46	6.54	6.25
**Kaempferol**	1.61	3.30	2.71	2.06	2.50	3.34	1.69	3.91	4.12	2.63	3.01	0.95	2.09	1.66	3.64	1.99
**Naringenin**	13.97	5.87	9.83	43.66	45.19	51.53	9.75	74.58	70.05	41.03	37.02	71.53	31.94	37.36	63.96	13.91
**Protocatechuic acid**	6.98	4.24	3.46	3.30	3.61	6.08	6.40	5.00	4.94	5.27	6.41	8.48	7.73	5.99	4.64	3.16
***p*-Hydroxybenzoic acid**	0.84	1.16	0.89	0.37	0.47	1.77	2.20	0.71	0.68	1.05	0.13	1.18	2.13	1.71	2.01	0.41
**Catechin**	2.78	2.83	2.76	2.68	2.83	2.61	2.86	2.88	2.87	2.67	2.74	2.74	2.84	2.77	2.81	2.68
***p*-Coumaric acid**	1.27	1.94	1.52	2.66	2.05	1.90	1.90	1.80	1.52	1.54	1.40	2.12	2.70	1.47	1.71	0.61
**Isorhamnetin-3-*O*-rutinoside**	1.81	1.84	1.79	1.74	1.84	1.69	1.86	1.87	1.86	1.73	1.78	1.78	1.85	1.80	1.82	1.74
**Ferulic acid**	2.61	2.66	2.59	2.52	2.66	2.44	2.68	2.70	2.69	2.50	2.57	2.57	2.67	2.60	2.63	2.52
**Kaempferol-7-*O*-glucoside**	2.49	2.54	2.47	2.41	2.54	2.34	2.56	2.58	2.57	2.40	2.46	2.46	2.55	2.49	2.52	2.41
**Apigenin**	0.14	0.09	0.14	0.19	0.20	0.29	0.10	0.44	0.39	0.19	0.27	0.26	0.14	0.18	0.24	0.10
**Luteolin**	2.92	2.28	2.22	1.44	2.44	4.17	2.82	5.32	4.45	3.25	6.42	4.14	1.72	1.46	3.95	0.89
**Eriodictyol**	2.31	2.35	2.29	2.23	2.36	2.17	2.37	2.39	2.38	2.22	2.28	2.28	2.36	2.30	2.33	2.23
**Gallic acid**	0.96	0.56	0.33	0.17	0.37	0.80	0.93	0.47	0.46	0.56	0.80	1.18	0.94	0.61	0.33	0.22
**Naringin**	1.45	1.47	1.44	1.40	1.48	1.36	1.49	1.50	1.49	1.39	1.43	1.43	1.48	1.44	1.46	1.40

1 – refer to the table 4. in article (as a list of collected cultivars).

2 – S- standard, R- resistant, A- autocthtonous type of cultivar.

3 – expressed in g/kg equavilents gallic acid.

4 – expressed in mmol/kg trollox equavilents.

5 – expressed in mg/kg (all individual compounds presented in the table).

Table S1. Continued

Tissue	Leaves															
**Year**	2018															
**Cultivar number**	16	17	18	19	20	21	22	23	24	25	26	27	28	29	30	31
**Type**	A	A	A	A	A	A	A	A	A	A	A	A	A	A	A	A
**TPC**	17.72	71.47	76.08	80.27	74.85	74.78	78.97	47.51	79.92	80.48	62.65	65.22	74.37	56.85	87.86	73.03
**RSA**	5.19	9.29	8.40	8.70	8.59	10.13	8.49	5.06	11.51	8.98	6.62	8.15	8.08	6.52	12.69	6.38
**5-*O*-Caffeoylquinic acid[5]**	23.52	105.99	168.18	175.81	56.20	122.64	157.90	94.25	44.75	145.69	132.38	80.78	184.12	159.70	104.65	143.92
**Caffeic acid**	0.30	1.81	4.85	3.40	4.21	2.33	3.25	0.75	3.57	4.49	2.36	2.17	2.85	2.47	2.55	1.64
**Phloretin**	85.56	148.19	178.15	228.82	655.07	238.57	202.48	240.48	320.92	209.66	215.30	287.57	199.46	213.93	266.58	186.21
**Phlorizin**	165.42	232.89	218.39	222.56	211.29	232.89	222.65	224.53	226.29	225.23	257.46	245.26	227.80	222.09	243.40	224.66
**Quercetin**	19.73	25.78	31.86	33.00	34.44	28.91	22.12	5.68	27.64	31.55	20.08	24.60	23.82	17.97	30.60	39.96
**Rutin**	58.48	77.08	141.80	172.23	72.02	75.62	145.62	31.68	54.40	158.64	95.33	132.07	162.45	37.69	83.44	138.70
**Quercetin-3-*O*-glucoside**	197.56	355.79	365.05	393.66	283.18	352.27	361.37	229.37	294.31	368.24	307.67	332.53	393.51	270.42	342.72	325.62
**Quercetin-3-*O*-rhamnoside**	105.73	173.48	225.15	241.02	180.33	162.53	234.54	89.10	212.36	254.42	137.11	184.13	240.26	120.73	232.45	179.48
**Isorhamnetin-3-*O*-glucoside**	6.25	6.68	6.18	6.31	6.10	6.49	6.24	6.69	6.39	6.47	6.62	6.71	6.44	6.45	6.22	6.05
**Kaempferol**	1.99	1.74	2.16	2.13	3.58	1.29	1.71	0.90	2.25	2.82	1.58	2.16	1.85	1.42	2.63	2.50
**Naringenin**	13.91	25.56	31.47	39.20	117.72	41.83	34.78	42.53	56.49	35.74	37.19	50.88	35.01	36.76	46.61	31.99
**Protocatechuic acid**	3.16	6.19	8.91	7.18	8.47	4.50	10.58	6.12	9.81	8.49	12.97	5.43	8.17	6.02	3.65	4.91
***p*-Hydroxybenzoic acid**	0.41	1.20	2.97	2.07	2.67	1.48	3.73	1.45	1.40	2.42	2.04	2.08	2.59	1.11	1.93	1.25
**Catechin**	2.68	2.86	2.65	2.71	2.62	2.78	2.68	2.87	2.74	2.77	2.84	2.88	2.76	2.77	2.67	2.60
***p*-Coumaric acid**	0.61	1.46	2.75	2.97	3.51	3.85	2.80	1.72	4.47	4.76	2.54	2.08	2.48	1.97	2.17	1.75
**Isorhamnetin-3-*O*-rutinoside**	1.74	1.86	1.72	1.76	1.70	1.81	1.74	1.87	1.78	1.80	1.85	1.87	1.80	1.80	1.74	1.69
**Ferulic acid**	2.52	2.69	2.49	2.54	2.45	2.61	2.51	2.69	2.57	2.60	2.67	2.70	2.59	2.60	2.50	2.44
**Kaempferol-7-*O*-glucoside**	2.41	2.57	2.38	2.43	2.35	2.50	2.40	2.58	2.46	2.49	2.55	2.58	2.48	2.49	2.40	2.33
**Apigenin**	0.10	0.14	0.16	0.17	0.39	0.19	0.17	0.24	0.23	0.16	0.22	0.22	0.19	0.19	0.21	0.21
**Luteolin**	0.89	2.17	2.55	2.30	6.65	2.40	2.40	1.78	3.96	3.23	2.16	2.75	1.98	2.04	2.30	3.07
**Eriodictyol**	2.23	2.38	2.20	2.25	2.18	2.31	2.23	2.39	2.28	2.31	2.36	2.39	2.30	2.30	2.22	2.16
**Gallic acid**	0.22	0.53	1.41	0.77	1.28	0.49	2.23	0.93	1.20	1.05	1.88	0.44	1.00	0.79	0.35	0.37
**Naringin**	1.40	1.49	1.38	1.41	1.36	1.45	1.39	1.49	1.43	1.44	1.48	1.50	1.44	1.44	1.39	1.35

1 – refer to the table 4. in article (as a list of collected cultivars).

2 – S- standard, R- resistant, A- autocthtonous type of cultivar.

3 – expressed in g/kg equavilents gallic acid.

4 – expressed in mmol/kg trollox equavilents.

5 – expressed in mg/kg (all individual compounds presented in the table).

Table S1. Continued

Tissue	Leaves														
**Year**	2019														
**Cultivar number**	1	2	3	4	5	6	7	8	9	10	11	12	13	14	15
**Type**	S	S	S	S	S	R	R	R	R	R	R	A	A	A	A
**TPC**	99.11	104.32	92.19	104.61	97.69	93.21	88.89	80.14	104.32	97.56	87.28	91.59	83.93	88.64	73.83
**RSA**	9.26	13.62	14.04	11.10	8.28	13.68	9.68	10.15	11.02	12.59	12.00	9.24	9.32	8.89	7.64
**5-*O*-Caffeoylquinic acid**[5]	112.33	72.55	97.00	70.45	59.14	78.24	89.20	103.18	72.44	75.41	43.34	132.30	200.04	242.98	116.82
**Caffeic acid**	1.88	2.16	2.06	2.24	0.67	3.96	2.80	2.91	1.34	2.72	0.92	0.70	1.39	0.63	0.08
**Phloretin**	188.52	51.72	126.03	90.88	140.59	497.00	105.71	597.85	220.90	122.93	161.32	269.55	135.03	213.52	242.57
**Phlorizin**	253.11	228.54	231.57	235.20	251.79	230.63	242.11	238.54	228.33	234.58	240.02	268.79	269.32	252.44	241.14
**Quercetin**	17.68	26.59	26.10	18.59	19.26	33.32	12.98	27.70	21.80	19.54	17.88	15.32	17.04	9.99	10.68
**Rutin**	92.10	159.94	115.69	122.18	81.83	144.17	138.99	97.31	98.87	80.60	86.36	84.34	125.94	60.74	216.52
**Quercetin-3-*O*-glucoside**	354.14	396.65	383.94	358.79	315.92	382.91	366.63	392.95	321.76	342.39	326.39	289.45	334.62	279.36	346.06
**Quercetin-3-*O*-rhamnoside**	173.90	243.92	200.04	207.81	200.54	274.48	191.40	253.26	283.70	325.70	190.68	104.11	218.95	116.53	203.14
**Isorhamnetin-3-*O*-glucoside**	6.11	6.62	6.05	6.10	6.34	6.25	6.59	6.43	6.51	6.65	6.71	6.15	6.61	6.47	6.14
**Kaempferol**	1.29	2.28	1.54	1.14	1.49	2.55	1.34	2.31	1.71	1.57	2.34	0.78	1.74	1.00	1.55
**Naringenin**	33.90	8.85	21.88	16.31	25.82	88.31	18.78	103.31	37.78	22.24	29.53	46.42	24.67	37.93	43.59
**Protocatechuic acid**	3.58	2.54	1.66	1.93	1.82	2.99	2.94	2.19	2.25	2.23	2.95	4.67	5.25	4.00	1.92
***p*-Hydroxybenzoic acid**	0.77	0.36	0.61	0.11	0.09	1.38	0.24	0.90	0.27	0.07	0.14	2.63	0.96	1.39	0.27
**Catechin**	2.62	2.84	2.60	2.62	2.72	2.68	2.83	2.76	2.79	2.85	2.88	2.64	2.84	2.78	2.64
***p*-Coumaric acid**	1.51	0.67	1.14	1.89	1.87	2.16	0.56	0.76	0.59	0.49	1.07	0.39	2.14	1.62	0.20
**Isorhamnetin-3-*O*-rutinoside**	1.70	1.85	1.69	1.70	1.77	1.74	1.84	1.79	1.81	1.85	1.87	1.71	1.84	1.80	1.71
**Ferulic acid**	2.46	2.66	2.44	2.45	2.55	2.51	2.65	2.59	2.62	2.67	2.70	2.47	2.66	2.60	2.47
**Kaempferol-7-*O*-glucoside**	2.35	2.55	2.33	2.35	2.44	2.41	2.54	2.48	2.51	2.56	2.58	2.37	2.54	2.49	2.37
**Apigenin**	0.12	0.10	0.10	0.08	0.08	0.38	0.10	0.35	0.18	0.13	0.20	0.13	0.07	0.14	0.14
**Luteolin**	2.14	1.96	1.68	1.90	1.87	4.05	2.46	3.55	3.08	3.61	6.34	2.09	0.87	1.42	1.16
**Eriodictyol**	2.18	2.36	2.16	2.18	2.26	2.23	2.35	2.29	2.32	2.37	2.39	2.19	2.36	2.31	2.19
**Gallic acid**	0.25	0.12	0.09	0.21	0.21	0.29	0.20	0.18	0.07	0.17	0.33	0.55	0.50	0.55	0.26
**Naringin**	1.37	1.48	1.35	1.36	1.42	1.40	1.47	1.44	1.45	1.49	1.50	1.37	1.48	1.45	1.37

1 – refer to the table 4. in article.

2 – S- standard, R- resistant, A- autocthtonous.

3 – expressed in g/kg equavilents gallic acid.

4 – expressed in mmol/kg trollox equavilents.

5 – expressed in mg/kg.

Table S1. Continued

**Tissue**	Leaves															
**Year**	2019															
**Cultivar number**	16	17	18	19	20	21	22	23	24	25	26	27	28	29	30	31
**Type**	A	A	A	A	A	A	A	A	A	A	A	A	A	A	A	A
**TPC**	82.71	100.83	97.17	114.02	87.64	94.29	75.30	97.00	94.84	90.64	93.30	90.62	83.16	82.25	121.22	83.39
**RSA**	7.12	8.61	7.90	8.15	10.31	9.36	5.23	11.15	8.31	7.54	9.41	7.82	8.02	8.29	13.97	7.71
**5-*O*-Caffeoylquinic acid**[5]	189.53	224.23	210.63	79.55	55.35	223.15	106.94	78.22	190.50	136.59	121.52	229.52	116.12	242.99	209.62	160.25
**Caffeic acid**	0.92	1.93	1.34	3.10	1.19	2.20	0.56	1.53	1.77	0.72	0.79	1.90	0.56	2.07	1.51	0.66
**Phloretin**	126.41	163.90	256.99	544.91	293.43	199.88	128.12	274.40	185.14	297.98	268.46	161.32	261.81	282.64	184.23	149.25
**Phlorizin**	240.02	266.35	264.01	272.20	251.33	252.47	262.87	247.11	248.61	265.01	280.90	264.56	233.19	253.84	290.45	269.54
**Quercetin**	7.25	19.52	10.60	18.80	14.26	15.26	3.85	10.76	12.66	14.35	13.87	13.06	19.12	12.59	15.35	5.80
**Rutin**	62.92	127.96	64.86	139.53	174.71	208.55	60.76	153.82	198.98	116.67	164.08	129.63	109.16	42.79	153.06	136.41
**Quercetin-3-*O*-glucoside**	286.96	351.21	311.45	307.52	354.91	390.78	247.85	340.41	365.74	286.16	320.22	358.97	351.30	260.74	378.47	310.27
**Quercetin-3-*O*-rhamnoside**	131.37	226.48	138.41	196.13	198.34	231.58	101.46	214.89	234.91	158.03	182.02	210.69	226.34	135.41	215.72	160.56
**Isorhamnetin-3-*O*-glucoside**	6.33	6.52	6.41	6.72	6.30	6.53	6.56	6.29	6.34	6.34	6.46	6.62	6.22	6.26	7.07	6.70
**Kaempferol**	1.04	1.61	0.89	1.27	0.75	1.32	0.94	1.44	1.17	1.67	2.12	1.68	2.28	1.66	1.58	1.40
**Naringenin**	22.62	29.44	46.52	98.54	51.25	34.15	22.56	48.41	32.64	53.14	48.59	28.57	46.33	49.35	33.64	25.81
**Protocatechuic acid**	3.59	5.09	2.46	4.96	5.34	4.41	3.01	3.97	3.82	6.84	2.80	5.26	1.62	4.44	3.78	3.28
***p*-Hydroxybenzoic acid**	0.51	0.68	0.96	2.50	1.36	1.13	2.35	2.33	1.35	1.99	2.08	0.94	0.59	1.11	1.48	1.70
**Catechin**	2.71	2.80	2.75	2.88	2.70	2.80	2.82	2.70	2.72	2.72	2.77	2.84	2.67	2.69	3.03	2.88
***p*-Coumaric acid**	1.26	1.96	1.65	4.27	1.36	0.93	0.39	2.36	2.89	2.07	1.67	2.57	1.43	2.00	2.16	1.86
**Isorhamnetin-3-*O*-rutinoside**	1.76	1.82	1.79	1.87	1.76	1.82	1.83	1.75	1.77	1.77	1.80	1.85	1.73	1.75	1.97	1.87
**Ferulic acid**	2.55	2.62	2.58	2.70	2.53	2.63	2.64	2.53	2.55	2.55	2.60	2.66	2.50	2.52	2.84	2.70
**Kaempferol-7-*O*-glucoside**	2.44	2.51	2.47	2.59	2.42	2.52	2.53	2.42	2.44	2.44	2.49	2.55	2.40	2.41	2.72	2.58
**Apigenin**	0.08	0.10	0.13	0.23	0.15	0.11	0.12	0.14	0.14	0.17	0.14	0.08	0.16	0.15	0.15	0.11
**Luteolin**	1.28	0.86	1.36	4.05	1.82	1.04	1.73	2.15	1.15	1.76	0.46	0.95	1.59	2.01	0.95	1.30
**Eriodictyol**	2.26	2.32	2.29	2.39	2.24	2.33	2.34	2.24	2.26	2.26	2.30	2.36	2.22	2.23	2.52	2.39
**Gallic acid**	0.41	0.63	0.27	0.63	0.70	0.41	0.28	0.58	0.52	0.81	0.33	0.65	0.04	0.53	0.59	0.27
**Naringin**	1.41	1.46	1.43	1.50	1.41	1.46	1.47	1.41	1.42	1.42	1.44	1.48	1.39	1.40	1.58	1.50

1 – refer to the table 4. in article (as a list of collected cultivars).

2 – S- standard, R- resistant, A- autocthtonous type of cultivar.

3 – expressed in g/kg equavilents gallic acid.

4 – expressed in mmol/kg trollox equavilents.

5 – expressed in mg/kg (all individual compounds presented in the table).

Table S2. Comparison of TPC values in different cultivar groups and years. Letters a-f denotes the grouping according to Tukey's HSD test at 0.95 confidence level.

Cultivar group	Sample	Range, year 2018 (mg/kg eq GA)	Range, year 2019 (mg/kg eq GA)
Standard	mesocarp	456 – 1413^{a,b}	100 – 734^{a,b}
	peel	3432 – 6508^{a,b}	9700 – 24892^{c}
	leaf	64794 – 88620^{d,f}	92191 – 104608^{e}
Resistant	mesocarp	333 – 640^{a,b}	125 – 703^{a,b}
	peel	3134 – 5444^{a,b}	7693 – 18368^{b,c}
	leaf	64117 – 78149^{d}	80136 – 104320^{e,f}
Autochthonous	mesocarp	737 – 4420^{a,b}	813 – 2591^{a}
	peel	4348 – 10591^{a,b}	18332 – 39054^{c}
	leaf	17724 – 87858^{d}	73834 – 121224^{e}

Table S3. Comparison of DPPH radical inhibition values in different cultivar groups and years. Letters a-d denotes the grouping according to Tukey's HSD test at 0.95 confidence level.

Cultivar group	Sample	Range, year 2018 (µmol/kg eq trolox)	Range, year 2019 (µmol/kg eq trolox)
Standard	mesocarp	8940 – 20969^{a,b,c}	7795 – 9644^{a,b}
	peel	29337 – 57636^{d,e}	23556 – 113338^{d,e}
	leaf	7557 – 11853^{a,b}	8282 – 14039^{a,b}
Resistant	mesocarp	7977 – 19732^{a,b}	7750 – 9579^{a,b}
	peel	26280 – 61208^{c,d,e}	21842 – 35450^{b,c,d}
	leaf	6403 – 11770^{a,b}	9683 – 11677^{a,b}
Autochthonous	mesocarp	8479 – 45523^{a,b}	6028 – 11912^{a,b}
	peel	29512 – 49346^{c,d,e}	23476 – 124545^{b,c,d}
	leaf	5056 – 12687^{a,b}	5231 – 12687^{a,b}

Table S4. Univariate test for significance of factor effects influencing total phenolic content (TPC) and DPPH radical scavenging activity (RSA) in the analysed samples. DF – Degrees of freedom; SS – Sum of squares; MS – Mean squares. Factors: F_1 – represents the three tissue types (mesocarp, peel and leaf), F_2 – describes the differences among the three apple cultivars (autochthonous, standard and resistant) and F_3 – is corresponding to the years of production.

		**TPC**				**RSA**			
**Factor**	*DF*	*SS*	*MS*	*F*	*p*	*SS*	*MS*	*F*	*p*
Intercept	1	1.35×10^{11}	1.35×10^{11}	2345.76	<0.001*	6.05×10^{10}	6.05×10^{10}	431.11	<0.001*
F_1	2	1.72×10^{11}	8.59×10^{10}	1495.48	<0.001*	2.73×10^{10}	1.37×10^{10}	97.35	<0.001*
F_2	2	2.09×10^{8}	1.04×10^{8}	1.82	0.166	6.26×10^{8}	3.13×10^{8}	2.23	0.111
F_3	1	4.77×10^{9}	4.77×10^{9}	82.92	<0.001*	1.03×10^{8}	1.03×10^{8}	0.74	0.392
$F_1 \times F_2$	4	6.88×10^{8}	1.72×10^{8}	2.99	0.020*	1.06×10^{9}	2.65×10^{8}	1.89	0.114
$F_2 \times F_3$	2	2.70×10^{9}	1.35×10^{9}	23.53	<0.001*	6.67×10^{8}	3.33×10^{8}	2.38	0.096
$F_2 \times F_3$	2	6.74×10^{7}	3.37×10^{7}	0.59	0.558	1.95×10^{8}	9.73×10^{7}	0.69	0.501
$F_1 \times F_2 \times F_3$	4	6.18×10^{7}	1.54×10^{7}	0.27	0.898	9.21×10^{8}	2.30×10^{8}	1.64	0.166

Statistically significant results at $p = 0.05$ are denoted with *

Table S5. Overview of detected compounds by OrbiTrap in mesocarp of the analyzed samples.

No	Compound name	Cultivar number[1,2]											
		2	3	9	11	12	13	14	15	16	17	18	19
1	Protocatechuic acid hexoside	+	+	+	+	+	+	+	+	+	+	+	+
2	Protocatechuic acid	+	+	+	+	+	+	+	+	+	+	+	+
3	Protocatechuic acid pentosylhexoside	+	-	+	-	-	-	+	+	+	-	+	+
4	Caffeoyl hexoside 1	+	+	+	-	+	+	+	-	-	+	+	+
5	*p*-Coumaric acid	+	+	+	-	+	+	-	-	+	+	+	+
6	Caffeoyl hexoside 2	+	+	+	-	-	+	+	+	+	+	+	+
7	Caffeoyl hexoside 3	+	+	+	+	+	+	+	+	+	+	+	+
8	5-*O*-Caffeoylquinic acid	+	+	+	+	+	+	+	+	+	+	+	+
9	*p*-Coumaric acid hexoside	+	+	+	+	+	+	+	+	+	+	+	+
10	5-*O*-Caffeoylquinic acid isomer	+	+	+	+	+	+	+	+	+	+	+	+
11	Methyl-3-*O*-caffeoylquinate	-	-	-	+	+	+	-	+	+	-	-	+
12	3-*p*-Coumaroylquinic acid	+	+	+	+	+	+	+	+	+	+	+	+
13	Methyl 5-*O*-caffeoylquinate	+	+	+	+	+	+	+	+	+	+	+	+
14	Caffeic acid	-	+	+	-	+	+	-	+	+	+	+	+
15	Methyl 3-*p*-coumaroylquinate	+	+	-	+	+	+	+	+	+	+	+	+
16	Methyl 5-*O*-caffeoylquinate isomer	+	+	+	+	+	+	+	+	+	+	+	+
17	Methyl 5-*p*-coumaroylquinate	+	+	+	+	+	+	+	+	+	+	+	+
18	Methyl 5-*p*-coumaroylquinate isomer	+	+	+	+	+	+	+	+	+	+	+	+
19	Rosmarinic acid	+	+	+	+	+	+	+	+	+	+	+	+
20	Caffeoyl-feruloylquinic acid	-	+	-	-	+	+	-	+	-	+	+	+
21	Prodelphinidin B type ((epi)gallocatechin-(epi)catechin)	+	+	+	+	+	+	+	+	+	+	+	+
22	(Epi)catechin-hexoside	+	+	-	-	+	+	-	+	-	+	+	+
23	Procyanidin B type 1 (catechin-(epi)catechin)	+	+	+	+	+	+	+	+	+	+	+	+
24	Epiafzelechin 3-*O*-gallate	+	+	+	+	+	-	+	+	+	+	+	+
25	Procyanidin B type 2 (catechin-(epi)catechin)	+	+	+	+	+	+	+	+	+	+	+	+
26	Catechin	+	+	+	+	+	+	+	+	+	+	+	+
27	Procyanidin B type 3 (catechin-(epi)catechin)	+	+	+	+	+	+	+	+	+	+	+	+
28	Epicatechin	+	+	+	+	+	+	+	+	+	+	+	+
29	(Epi)catechin-methyl(epi)gallocatechin	+	+	+	+	+	+	+	+	+	+	+	+
30	Taxifolin	+	+	-	-	+	+	+	-	+	+	+	+
31	Quercetin 3-*O*-rhamnoside	+	+	+	+	+	+	+	+	+	+	+	+
32	Kaempferol 3-*O*-pentoside	+	+	-	-	-	-	-	-	+	-	+	+
33	Kaempferol 3-*O*-rhamnoside	+	+	+	+	+	+	+	-	-	+	+	+
34	Phloretin 2′-*O*-(2′′-*O*-pentosylhexoside)	+	+	+	+	+	+	+	+	+	+	+	+
35	Phloretin	+	+	+	+	+	+	+	+	+	+	+	+
36	Phloretin 2′-O-hexoside (Phloridzin)	+	+	+	+	+	+	+	+	+	+	+	+

1 – refer to the table 4. in article (as a list of collected cultivars)

2 – the detected compound is denoted with +, otherwise with -

Table S5. continued

No	Compound name	Cultivar number[1,2]											
		20	21	22	23	24	25	26	27	28	29	30	31
1	**Protocatechuic acid hexoside**	+	+	+	+	+	+	+	+	+	+	+	+
2	**Protocatechuic acid**	+	+	+	+	+	+	+	+	+	+	+	+
3	**Protocatechuic acid pentosylhexoside**	+	-	+	+	-	+	+	-	+	+	+	+
4	**Caffeoyl hexoside 1**	+	-	+	+	-	+	+	-	+	+	+	+
5	***p*-Coumaric acid**	+	+	-	+	+	+	+	+	+	+	+	+
6	**Caffeoyl hexoside 2**	+	+	+	+	+	+	+	+	+	+	+	+
7	**Caffeoyl hexoside 3**	+	+	+	+	+	+	+	+	+	+	+	+
8	**5-*O*-Caffeoylquinic acid**	+	+	+	+	+	+	+	+	+	+	+	+
9	***p*-Coumaric acid hexoside**	+	+	+	+	+	+	+	+	-	+	+	+
10	**5-*O*-Caffeoylquinic acid isomer**	+	+	+	+	+	+	+	+	+	+	+	+
11	**Methyl-3-*O*-caffeoylquinate**	-	-	-	-	-	-	+	-	-	-	-	-
12	**3-*p*-Coumaroylquinic acid**	+	+	+	+	+	+	+	+	+	+	+	+
13	**Methyl 5-*O*-caffeoylquinate**	+	+	+	+	+	+	+	+	+	+	+	+
14	**Caffeic acid**	+	+	-	+	+	+	+	+	+	+	+	+
15	**Methyl 3-*p*-coumaroylquinate**	+	+	+	+	+	+	+	+	+	+	+	+
16	**Methyl 5-*O*-caffeoylquinate isomer**	+	+	+	+	+	+	+	+	+	+	+	+
17	**Methyl 5-*p*-coumaroylquinate**	+	+	+	+	+	+	+	+	+	+	+	+
18	**Methyl 5-*p*-coumaroylquinate isomer**	+	+	+	+	+	+	+	+	+	+	+	+
19	**Rosmarinic acid**	+	+	+	+	+	+	+	+	+	+	-	+
20	**Caffeoyl-feruloylquinic acid**	+	+	-	+	+	+	+	+	-	+	-	+
21	**Prodelphinidin B type ((epi)gallocatechin-(epi)catechin)**	+	+	+	+	+	+	+	+	+	+	+	+
22	**(Epi)catechin-hexoside**	+	+	-	+	+	+	+	+	+	+	+	+
23	**Procyanidin B type 1 (catechin-(epi)catechin)**	+	+	-	+	+	+	+	+	+	+	+	+
24	**Epiafzelechin 3-*O*-gallate**	+	+	+	+	+	+	+	+	+	+	+	+
25	**Procyanidin B type 2 (catechin-(epi)catechin)**	+	+	+	+	+	+	+	+	+	+	+	+
26	**Catechin**	+	+	+	+	+	+	+	+	+	+	+	+
27	**Procyanidin B type 3 (catechin-(epi)catechin)**	+	+	+	+	+	+	+	+	+	+	+	+
28	**Epicatechin**	+	+	+	-	+	+	+	+	+	+	+	-
29	**(Epi)catechin-methyl(epi)gallocatechin**	+	+	+	+	+	+	+	+	+	+	+	+
30	**Taxifolin**	+	+	+	-	+	+	+	+	+	+	+	+
31	**Quercetin 3-*O*-rhamnoside**	+	+	+	+	+	+	+	+	+	+	+	+
32	**Kaempferol 3-*O*-pentoside**	-	-	-	+	-	+	+	-	-	+	+	+
33	**Kaempferol 3-*O*-rhamnoside**	+	-	+	+	-	+	+	+	-	+	+	+
34	**Phloretin 2′-*O*-(2′′-*O*-pentosylhexoside)**	+	+	+	+	+	+	+	+	+	+	+	+
35	**Phloretin**	+	+	+	+	+	+	+	+	+	+	+	+
36	**Phloretin 2′-O-hexoside (Phloridzin)**	+	+	+	+	+	+	+	+	+	+	+	+

1 – refer to the table 4. in article (as a list of collected cultivars)

2 – the detected compound is denoted with +, otherwise with -
